# Two strategies to engineer flexible loops for improved enzyme thermostability

**DOI:** 10.1038/srep41212

**Published:** 2017-02-01

**Authors:** Haoran Yu, Yihan Yan, Cheng Zhang, Paul A. Dalby

**Affiliations:** 1 Department of Biochemical Engineering, University College London, Gordon Street, London, WC1H 0AH, United Kingdom

## Abstract

Flexible sites are potential targets for engineering the stability of enzymes. Nevertheless, the success rate of the rigidifying flexible sites (RFS) strategy is still low due to a limited understanding of how to determine the best mutation candidates. In this study, two parallel strategies were applied to identify mutation candidates within the flexible loops of *Escherichia coli* transketolase (TK). The first was a “back to consensus mutations” approach, and the second was computational design based on ΔΔ*G* calculations in Rosetta. Forty-nine single variants were generated and characterised experimentally. From these, three single-variants I189H, A282P, D143K were found to be more thermostable than wild-type TK. The combination of A282P with H192P, a variant constructed previously, resulted in the best all-round variant with a 3-fold improved half-life at 60 °C, 5-fold increased specific activity at 65 °C, 1.3-fold improved *k*_cat_ and a *T*_m_ increased by 5 °C above that of wild type. Based on a statistical analysis of the stability changes for all variants, the qualitative prediction accuracy of the Rosetta program reached 65.3%. Both of the two strategies investigated were useful in guiding mutation candidates to flexible loops, and had the potential to be used for other enzymes.

Transketolase (TK), a thiamine diphosphate dependent (ThDP) enzyme, catalyses the reversible transfer of a C2-ketol unit from D-xylulose-5-phosphate to either D-ribose-5-phosphate or D-erythrose-4-phosphate, linking glycolysis to the pentose phosphate pathway in all living cells^1^,^2^. The stereospecifically controlled carbon-carbon bond forming ability of TK makes it promising as a biocatalyst in industry, for the synthesis of complex carbohydrates and other high-value compounds^3^,^4^. The use of β-hydroxypyruvate (HPA) as the ketol donor renders the donor-half reaction irreversible, thus increasing the atom efficiency of the reaction favourably for industrial syntheses. *Escherichia coli (E. coli)* TK converts HPA with a rate of 60 U/mg, significantly higher than the rates of 2 U/mg and 9 U/mg reported for its orthologs from spinach and yeast^5^.

Wild-type (WT) *E. coli* TK has been successfully engineered to have improved and inverted enantioselectivity^6^, as well as an expanded aldol-acceptor substrate range including polar aliphatics^7^, non-polar aliphatics^8^, and heteroaromatics^9^,^10^. Most recently, *E. coli* TK has been engineered to synthesize L-*gluco*-heptulose from L-arabinose, thus transforming a major component of the carbohydrates in sugar beet pulp, into a rare naturally-occurring ketoheptose with potential therapeutic applications in hypoglycaemia and cancer^11^. Additionally, the substrate range accepted by TK has been recently extended to aromatic benzaldehyde derivatives, which opened up potential routes to chiral aromatic amino-alcohols such as chloramphenicol antibiotics, nor-ephedrine, and their analogues with alternative aromatic substituents^12^,^13^.

As a mesophilic enzyme *E. coli* TK suffers the limitation of low stability to elevated temperatures and extremes of pH^14^, limiting its current use in industrial processes. High temperatures are often used to enhance reaction rates, increase reactant solubility, and decrease the risk of microbial contamination. *E. coli* TK has a broad optimum activity at 20–40 °C and loses activity rapidly at above 55 °C due to irreversible aggregation^14^. It therefore remains a challenge to design efficient bioconversions of aliphatic or aromatic aldehyde substrates by *E. coli* transketolase, at elevated temperatures to enhance their solubility in water. In addition, limited enzyme stability can be a barrier to further improvements in activity by mutagenesis.

Our recent mutagenesis of cofactor-binding loops towards those amino-acids found in *Thermus thermophilus* at equivalent positions, provided some success in which the H192P variant increased the optimal temperature for activity from 55 °C to 60 °C, with a linked increase in the *T*_agg_ measured by dynamic light scattering, from 60 °C to 61.5 °C^15^. However, considerable improvement is still required in both the specific activity and the half-life of the enzyme at elevated temperatures, to achieve good conversion yields.

Various techniques have been applied to enhance the thermostability of enzymes, including protein engineering, post-translational enzymatic or chemical modification, use of additives, and immobilization^16^. Targeted mutagenesis guided by structural or sequence information has emerged as a popular protein-engineering route for altering various properties of enzymes. Unlike traditional directed evolution consisting of iterative cycles of library construction using random mutagenesis and high-throughput screening, this strategy focuses on several specific mutations sites such that no high-throughput screening approach is required^17^. Targeted mutagenesis is less likely to disrupt the global protein fold and thus increases the probability for obtaining active variants. The focus on specific amino-acid positions significantly reduces the size of generated libraries and subsequently increases the efficiency of directed evolution, provided that the target-site prediction is reliable^18^.

One potential target for protein stabilisation is their flexible loop regions. Loops are a diverse class of structures including both well-defined turns and more disordered random coils, which generally connect the main secondary structures, α-helices and β-strands^19^. Loops are important structural elements in proteins, often associated with a higher sequence variability across homologs, thus contributing to diversification in terms of function within different clades of the same superfamily^20^. A growing number of studies have shown that loops play a significant role in modulating enzyme catalysis^21^, specificity^22^, stability^23^, and protein-protein interactions^24^. Therefore, loops might be good targets for engineering enzymes with newly acquired or improved properties. Several enzymes have been successfully engineered to have altered stability by carrying out mutations in loop regions^25^,^26^.

Considerable attention has, recently, been given to the role of fluctuations in protein thermostability. Highly fluctuating regions are believed to have a relatively low number of contacts with other amino acids, while large thermal fluctuations within flexible regions potentially expose the hydrophobic core of a protein to water penetration, triggering protein unfolding^27^. The assumption that rigidity is a prerequisite for high thermostability was supported by studies that compared mesophilic and thermophilic proteins^28^,^29^. However, other experimental and computer simulation studies imply that thermal tolerance is not necessarily correlated with the suppression of internal fluctuations for all proteins^30^.

With flexible sites as targets, a number of enzymes have been engineered to have improved thermostability^31^. B-Factor analysis and molecular dynamics (MD) simulation are two commonly used methods to investigate protein flexibility. As an experimental approach, B-Factors are obtained from X-ray data which indicate smearing of atomic electron densities with respect to their equilibrium position^32^. Since B-Factors are dependent on the resolution of crystal structures used, it is difficult to compare them from proteins with different resolution, unless structures at similar resolution are employed. MD simulation focuses on the dynamic motion of proteins during a simulated period of time, and provides accurate representations of protein flexibility under physiological-like environments. However, compared to B-Factor analysis, MD simulations are more time-consuming. Flexible sites could be engineered by various approaches such as iterative saturation mutagenesis^33^, RosettaDesign^34^, the introduction of disulphide bonds or prolines^35^, or the addition of salt bridges^36^ although no single approach is consistently successful at increasing thermostability.

The evolved enzymes guided by the RFS (rigidifying flexible sites) strategy tend to maintain a comparable catalytic activity with that of wild type, mainly because the most flexible tend to be located on the protein surface, far away from catalytic sites. However, in one recent exception, the introduction of disulfide bonds to flexible sites was used to engineer *Candida antarctica* lipase B (CalB) for enhanced thermostability. A variant N169C-F304C showed an improved conformational stability but a decreased thermal deactivation. Investigation of conformational change at molecular level indicated that the catalytic sites were influenced by the mutations, although the formed disulfide bond rigidified the flexible regions^35^. The relationship between flexibility, stability, and activity can therefore be complex. Rigidity is needed to maintain integrity of the native folded structure, whereas a certain degree of flexibility is required for activity. The number of cases successfully employing the RFS strategy is still limited mainly due to a limited understanding of how to determine the best mutation candidates.

Here we aimed to improve the thermostability of *E. coli* TK using a targeted mutagenesis approach. Flexible loops were selected as the mutation targets, and then two parallel strategies were applied to identify mutation candidates within those loops. The first was a “back to consensus mutations” approach^37^, and the second was computational design based on ΔΔ*G* calculations in Rosetta^38^. Forty-nine single-mutant variants and one double-mutant variant were generated and assessed for their impact on catalytic activity and thermostability. From these, three single-variants and one double-variant were found to be more thermostable than wild-type TK. The best variant had a 3-fold improved half-life at 60 °C, and an increase in *T*_*m*_ of 5 °C above that of wild type. We confirmed that flexible loops could be selected as “hot spots” for engineering protein thermostability, and that thermostability is greatly correlated to rigidity.

## Results and Discussion

### Identification of flexible and thermally-sensitive loops in TK

The PyMol molecular graphics system (Schrödinger, USA) was used initially to highlight secondary structure as annotated within the pdb file of TK (PDB ID 1QGD). A total of 39 loops were identified with the longest one, loop5 90–117 containing 26 amino acids and the shortest ones like loop3, only containing 2 amino acids (Supplementary Table S1). Here, with the TK 3D crystal structure (PDB: 1QGD) as input, the average B-Factor for each residue was calculated with the B-FITTER program, and the B-Factor for each loop was calculated by averaging the B-Factors of all residues within the loop. In order to understand the relationship between flexibility and the location of loops, the depth of loops was also calculated using the DEPTH server.

**Figure 1:**
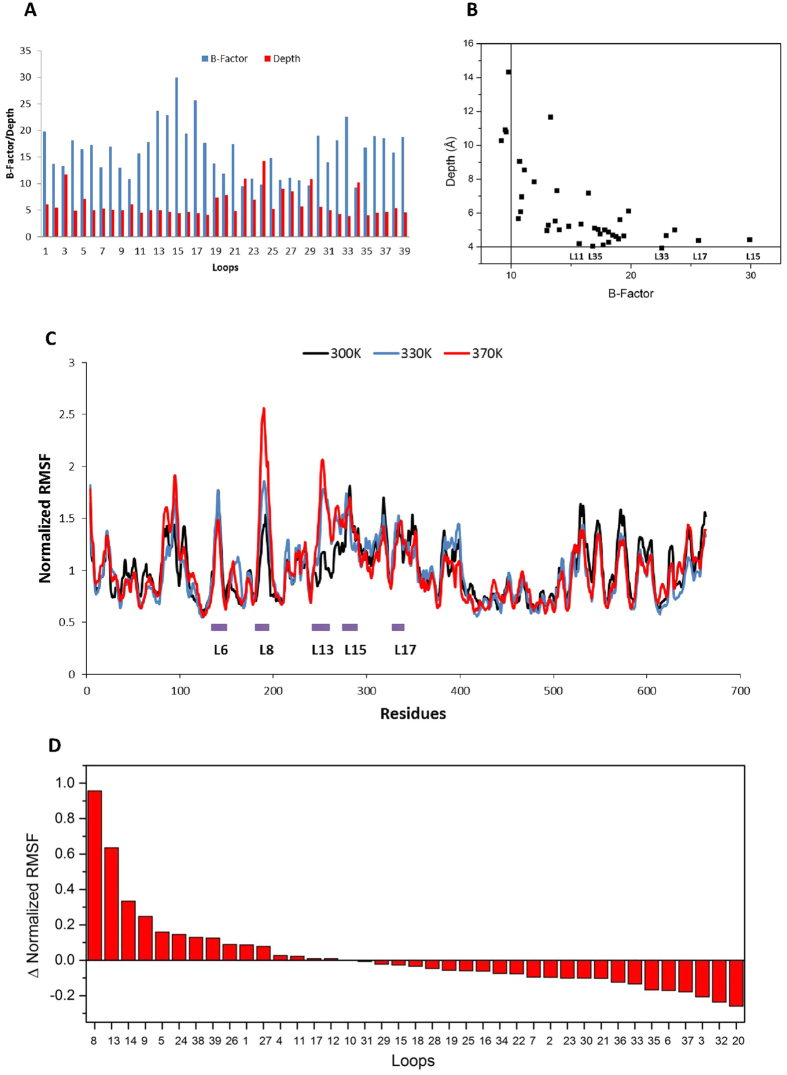
Flexibility and depth of loops in TK. (**A**) Comparison of B-Factor and depth of 39 loops in TK. The Y-axis is for both B-Factor and Depth (Å). (**B**) Correlation of B-Factor and depth for loops. (**C**) Normalized RMSF values versus residues of TK at different temperatures. (**D**) Difference of normalized RMSF values between 370 K and 300 K for each loop.

As expected, loops with higher flexibility tended to locate at the protein surface, whereas loops deeply buried in the protein tended to have lower flexibility than surface loops (Fig. 1). However, some exceptions were observed. Loop3 is interesting as it has a relatively high B-Factor for its depth. Only containing two residues Ser63 and Asn64, loop3 is a small loop located in the active site, and close to the dimer interface. Asn64 interacts sterically with the ThDP cofactor, and also with catalytic residue His66 which is directly involved in the substrate specificity of the TK (Supplementary Fig. S1). Given that loop3 is located quite deeply within the protein, its relatively high flexibility may be required for efficient enzyme catalysis.

Loops11, 15, 17, 33, 35 are all located on the protein surface with similar depths of around 4 Å, but their average B-Factors varied greatly, from 15.7 to 29.9. Flexibility is an indicator of the accumulation of interactions. Since these loops are located at a similar depth, their differences in flexibility will be heavily determined by the number (per residue), type and strength of interactions they make. Salt bridges and hydrogen bonds were analysed for loops11, 15, 17, 33, 35, and listed in Supplementary Table S2. Loop11 214–220 is the most rigid of these five surface loops, with a B-Factor of 15.7, and contained the most interactions. Three salt bridges and 12 hydrogen bonds were found to rigidify this loop. Similarly, the salt bridge Asp545-Arg579 within loop33 could be the reason for its lower flexibility than loop17. Loop33 contains the same number of amino acids but fewer hydrogen bonds than loop17, suggesting that the salt bridge contributes more to rigidifying the loop than the four additional hydrogen bonds in loop17. However, as the total number of hydrogen bonds is higher than the number of salt bridges in proteins, their accumulative contribution to protein stability cannot be neglected. For example, loop35 592–593 was relatively rigid as a result of seven hydrogen bonds found in this small two-residual loop.

Molecular dynamics (MD) simulations were used to investigate flexibility independently from B-Factors. A 30-ns molecular dynamics simulation was applied to analyse the flexibility of the wild-type TK structure. The backbone RMSD remained at around 0.13 nm and that the structure became relatively stable within 15 ns of a 30 ns molecular simulation performed at 300 K (Supplementary Fig. S2A). Based on the results of RMSD, the last 10 ns of the trajectory was selected to calculate the RMSF (root-mean-square fluctuation) values for all atoms except hydrogen in each residue. However, since the baseline values of average RMSF varied greatly between runs (Supplementary Fig. S2B), the original RMSF values were normalized by the average RMSF of the whole protein. We compared the normalized RMSF values of each residue with the average B-Factor values obtained from the crystal structure to validate its performance (Supplementary Fig. S3). Although B-factor values and RMSF values reflect protein flexibility in different aspects, they have significant similarity (Supplementary Fig. S3), with a Pearson’s correlation coefficient of 0.83, indicating that RMSF is consistent with B-Factors in the prediction of protein flexibility. However, despite the strong correlation, there were differences worth noting. For example, the five most flexible loops were loop15, loop6, loop33, loop17 and loop8 based on RMSF values calculated from an MD trajectory run at 300 K (Fig. 1C), whereas the five most flexible loops were loop15, loop17, loop13, loop14 and loop33 predicted by B-Factors (Supplementary Table S1).

MD simulations performed at higher temperatures could potentially give more information relating to protein unfolding. Two additional simulations of TK were performed at 330 K and 370 K to identify thermally sensitive regions. As shown in Fig. 1C, the pyrophosphate (PP)-binding domain (2–322 aa) and the C-terminal domain (540–663 aa) were more flexible than the pyrimidine (Pyr)-binding domain (323–539 aa). Residues 400–500 within the Pyr domain showed the lowest fluctuation under all three temperatures, suggesting that this highly buried region is also relatively thermostable. The difference in normalized RMSF values between 300 K and 370 K shows that several loops exhibited steep changes in fluctuation as a function of temperature (Fig. 1D).

The most thermally sensitive loop was loop8, one of two cofactor-binding loops. A previous study has shown that deactivation, denaturation and aggregation of TK at extreme pH or high temperatures is strongly linked to the binding of cofactors, and to the structure of the cofactor-binding loops^14^. More importantly, this loop has been engineered by mutating it towards the equivalent loop of a thermostable orthologue, *Thermus thermophilus*, and the best variant, H192P, showed both improved activity and stability indicating the importance of thermally sensitive loops in regulating protein stability^39^. By contrast, the other cofactor-binding loop, loop21 in this study, did not show an increase in flexibility as the temperature was increased (Fig. 1D and S4). Consistent with this observation, previous mutations tested in this loop did not lead to any improvements in thermostability^39^. As observed for loop21, several loops including loops32–37, led to a decrease in normalized RMSF values at higher simulation temperatures. These loops tended to locate deeply in the protein (See Fig. 1A), consistent with deeply buried residues having a stronger tolerance to thermal stress compared to surface residues.

Although loop8 was previously shown to be a critical region in which to engineer the thermostability of TK, this loop failed to be detected by the B-Factor approach which ranked loop8 at only eighteenth among the 39 loops (Supplementary Table S1), indicating a potential limitation of using the B-Factor approach in isolation for predicting flexible regions of a protein. One of the reasons that the B-Factor approach underestimated the flexibility of loop8 could be the presence of the cofactor ThDP in the crystal structure (1QGD.pdb). It is known from a comparison of the crystal structures of holo-TK and apo-TK^40^, that the cofactor-binding loops are non-structured in the absence of ThDP, but become structured upon ThDP binding. It is thus important to consider the B-Factor values both in the presence and absence of such ligands when relying upon that approach alone. In addition, protein flexibility in solution might differ qualitatively from that in a crystal and the possible reasons have been discussed previously^31^.

Despite the potential pitfalls, the B-FIT approach^33^ has been used successfully to engineer the thermostability of several enzymes such as lipase from *Yarrowia Lipolytica*^41^, lipoxygenase from *Anabaena* sp. PCC 7120^42^ and esterase from *Pseudomonas fluorescens*^43^. Being aware of the limitation of B-Factors, it is beneficial to apply a complementary method like MD simulation to investigate protein flexibility. Considering both methods for TK, we finally selected loop6, loop8, loop13, loop15 and loop17 as five targets for engineering the thermostability of TK (Fig. 1C and S5). Loops13, 15 and 17 were predicted to be flexible by both MD simulation and B-Factors, loop6 was picked based on RMSF values at 300 K, and loop8 was selected based on the largest ΔRMSF due to the increased MD simulation temperature.

### Introduction of mutations guided by the consensus concept

Amino acids appearing at a specific position most frequently among homologous structures, contribute to the stability more than other residues at the same position. Based on this assumption, the “consensus design” approach has been widely used in engineering protein stability. A related and simple approach is to compare the sequence or structure of the target enzyme with that of homologs from thermophilic organisms, to identify key sites for mutagenesis. In this way the two co-factor loops of *E. coli* TK, have been mutated previously toward those of *Thermus thermophilus* TK. A single variant H192P gave a two-fold improved stability to inactivation at elevated temperature, and three-fold improved specific activity compared to WT at 60 °C^15^.

Loops include both well-defined turns and disordered random-coil-like structures. The β-turn is a well-defined turn, consisting of 4 residues (positions i, i + 1, i + 2, i + 3) and has been classified into nine different types based on the dihedral angle values of the i + 1 and i + 2 position in the turn^44^. Although poor amino-acid consensus has been observed in loop regions, a statistical analysis of residues constituting 7153 β-turns of 426 protein chains showed that significant residue preferences occur at specific β-turn positions^44^. For example, four residues (A, E, K, P) occur with higher frequency than others at the second position of type II β-turns. From 911 type II β-turns, 213, 95, 89, and 76 respectively had proline, alanine, lysine, glutamic acid at the second position. According to the consensus concept, if the residue in the second position of a type II β-turn is not A, E, K or P, then mutating it to one of them might improve the stability of the target protein. Using this assumption we designed 40 single variants within the selected flexible loops, with the aim of improving the stability of TK. Loop8 was ruled out in this strategy as it had already been engineered previously to the consensus, using the H192P mutation.

The β-turn locations within TK (1QGD.pdb) were obtained from the PDBsum database (Supplementary Table S3). There were 47 isolated or overlapping β-turns in transketolase, all of which belonged to four types I, II, IV, VIII. In this study, we only considered type I and II β-turns since they represent the majority of the different types of known β-turns and showed previously the most statistically significant amino-acid positional preferences^44^. Overlapping β-turns are those that share one or more residues with other β-turns such as turns 20–24, all of which locate in loop13 245–257. Such overlapping turns appear to be common in proteins, and comprise 58% of all β-turns^45^. For overlapping β-turns, we only considered the overlapped residues when designing potential mutants. Since turns 22, 23 and 24 shared residue Lys254, it was mutated to C, G, N, D, S, T, P based on the sequence statistics of β-turns (Supplementary Fig. S6, Table 1). In addition to three overlapping β-turns, an isolated β-turn 246IIGF249 was also detected in loop13. As a type II β-turn, the amino acid found at the first position in wild-type *E. coli* TK was isoleucine, which was not in agreement with the statistically preferred C or P (Supplementary Fig. S6). Therefore, I246C, and I246P were selected as mutation candidates. Following this strategy, all possible mutants in loops6, 13, 15, 17 were designed and shown in Table 1.

**Figure 2:**
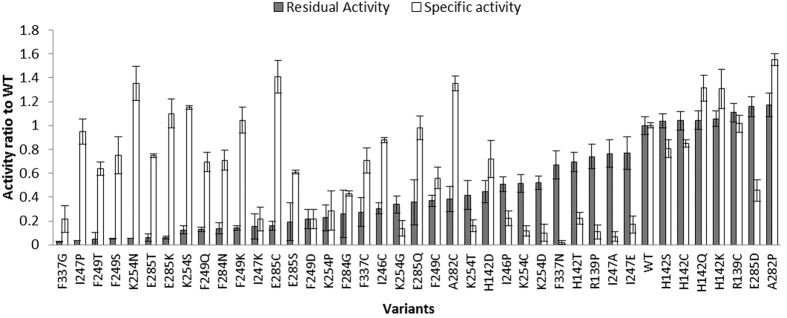
Residual activity and specific activity for 40 consensus variants relative to wild-type TK. Residual activity was measured by incubating 200 μL cell lysates in triplicate under 60 °C for 1 h. Specific activity was calculated by dividing initial activity by OD_600_ of culture.

The activity and stability of all generated variants were investigated initially using a microplate-based screening method. Specific activity and residual activity ratios relative to WT were calculated for comparison, and the variants ranked based on the relative residual activity (Fig. 2). Most of the variants decreased the thermostability relative to WT, except for two variants in loop15, A282P and E282D, and five variants in loop6, H142C/K/Q/S, and R139C. Although these seven variants did not all show a significant improvement of stability according to the screening conditions, they mostly maintained specific activities comparable to WT. In addition, some of the variants such as K254D, R139P, and I247K led to a significant loss of specific activity. Nineteen variants were designed within loop13 245–257 (Table 1) and, surprisingly, most of them showed both decreased activity and stability. Loop13 is near to an invariant active-site residue His261, which interacts with the diphosphate of ThDP and the C3-hydroxyl groups of the ketol donor substrate, and forms part of the active-site funnel leading to the co-factor ThDP and substrates.

To evaluate the true effect of mutations on the properties of TK, we purified several variants showing relatively high residual activity, and compared their thermostabilities with the wild type, but first we adopted a second computational approach for additional mutant designs as below.

### Computational design of variants using Rosetta

Several algorithms have been developed to predict protein stability changes due to mutations, ΔΔ*G*, for which negative values represent stabilising mutations. A recent comparison indicated that Rosetta ddg_monomer program generally provided more accurate results than three other methods^38^,^46^. To investigate whether ΔΔ*G* prediction is an effective strategy to guide mutation candidates in flexible loops, we calculated the ΔΔ*G* values of all possible single substitutions in the flexible loops of TK using the Rosetta ddg_monomer program.

**Figure 3:**
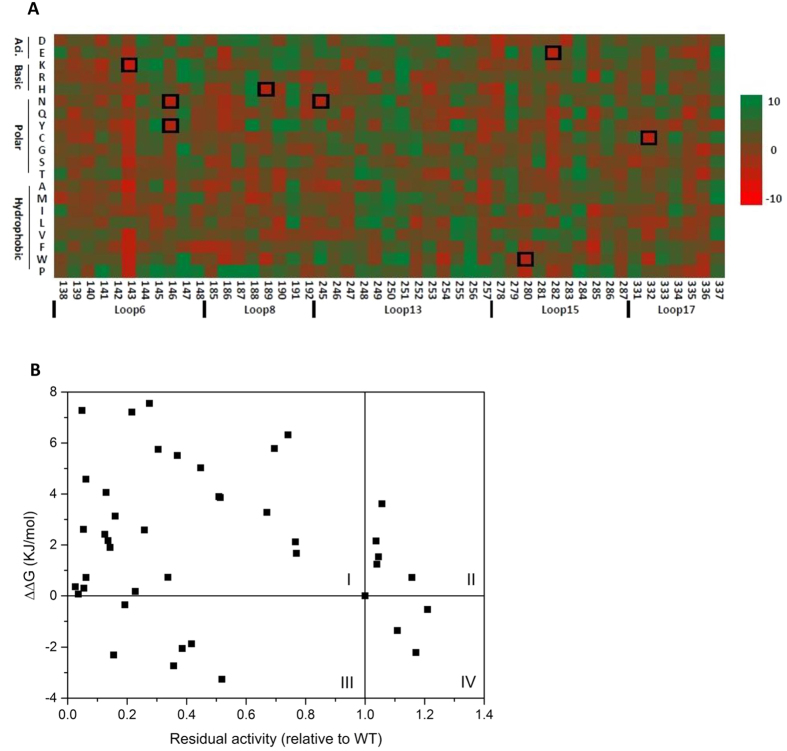
Stability prediction of TK variants by Rosetta. (**A**) Heat map of ΔΔ*G* values for TK variants showing stable (red) and unstable (green) variants. Black squares highlight the variants generated and tested experimentally. (**B**) Correlation between ΔΔ*G* values and residual activity (relative to wild type) of 41 TK consensus variants in lysates.

There were 49 amino acids in the flexible loops (loops6, 8, 13, 15, 17) and 931 possible single variants in total. A heat map of all 931 predicted ΔΔ*G* values suggested that most substitutions would be neutral or deleterious (Fig. 3A). The positions predicted to tolerate very little sequence variation include Gly191 in loop8, Gly248-Ser251 in loop13, and Phe337 in loop17. In the consensus approach above, we already constructed several variants at positions Phe249 and Phe337. All of these variants F249C/D/K/Q/S/T, and F337C/G/N failed to improve the stability of TK, which agreed with Rosetta’s prediction. As for Gly191, this was already the consensus residue in an alignment of 54 homologous TK sequences.

Asp143 in loop6, was predicted to be tolerant of mutation to all amino acids except leucine, suggesting its potential to be a hotspot for engineering the stability of TK (Fig. 3A). The heat map of predicted ΔΔ*G* values also showed some amino acid preferences at certain positions. For example, Val145, also in loop6, was predicted to be tolerant only to leucine and isoleucine. These are both larger hydrophobic residues than valine, indicating the potential to optimise hydrophobic packing in this region.

To investigate the accuracy of the Rosetta ddg_monomer program, we compared the predicted ΔΔ*G* values with the residual activities in lysates, obtained above for the 41 consensus variants, including the previously identified thermostable variant, H192P in loop8 (Fig. 3B). There was no obvious quantitative correlation between predicted ΔΔ*G* values and their residual activity. However, the data in Fig. 3B was clustered into two main groups, each with an inverse correlation between residual activity and predicted ΔΔ*G*. The group with the lowest residual activity was formed by mutations that were clustered near the active site. By contrast the mutations with greater residual activity were distributed throughout the PP-domain both within and far from the active site, and also with one mutational site in the PYR domain. The flexible residues targeted in the cluster close to the active site, appear to become more prone to thermal inactivation upon mutation than variants in the other cluster, which indicates an additional mechanism of destabilisation that is not accounted for within the global ΔΔ*G* calculation. For example, the region may be particularly sensitive to introducing irreversible local unfolding, or to increasing the local aggregation propensity, upon mutation.

Qualitatively, the variants can be divided into four zones with the WT located at the cross centre (Fig. 3B). The majority of the variants were predicted to be destabilising, and this is consistent with the experimental results. Qualitatively, the 30 variants in zone I and IV (73.2%) were predicted correctly by Rosetta, while the 11 variants in zone II and III (26.8%) were not. The variant H192P is located in zone IV with the ΔΔ*G* value of −0.537, significantly higher than the lowest ΔΔ*G* value of D143K (−6.384), although its effect on stability was predicted correctly by Rosetta.

The assessment of thermostability as a residual activity after heating, does not necessarily reflect the stability of a purified protein, as the lysate environment could influence the conformational stability and also the aggregation rate of and enzyme. Furthermore, differences in expression levels in lysates between variants could also influence the aggregation rates as this is concentration dependent. Hence, to further investigate the accuracy of the Rosetta ddg_monomer program in predicting stable variants independently from the consensus approach, we generated, purified and tested the thermostability of the 8 variants with the lowest predicted ΔΔ*G* values.

### Characterisation of purified variants with greater stability

**Figure 4:**
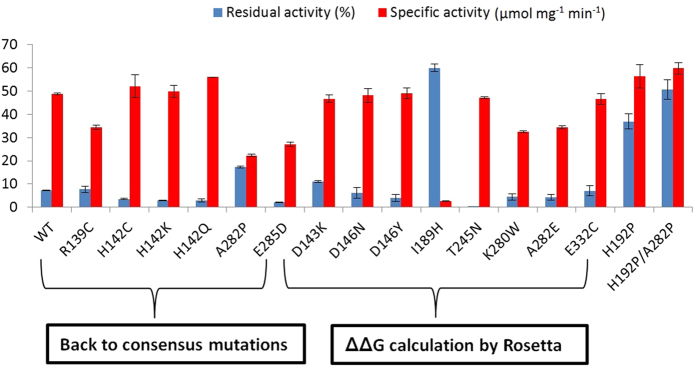
Thermostability and specific activity of TK variants from two strategies. Thermostability was examined by incubating 100 μL 0.1 mg/mL enzymes in triplicate under 60 °C for 1 h. Enzyme activity was measured at 22 °C with 50 mM GA, 50 mM HPA, in 50 mM Tris-HCl, pH 7.0 before and after heating. Specific activity in the plot was the initial activity measured before heating.

We generated and purified 6 top hits from the consensus approach, and the 8 best Rosetta designs from above (14 variants in total). Of the variants guided by the “back to consensus” concept, A282P improved the residual activity 2.4-fold compared to wild type, whereas the other five variants retained a similar or lower activity after heat inactivation (Fig. 4). As H192P was constructed previously based on a related consensus strategy, and found to improve the residual activity 5-fold above wild type, we further combined H192P and A282P to get the double variant H192P/A282P. This variant was found to be more stable than both H192P and A282P, with a 7-fold improvement in residual activity compared to wild type, and retained 50% activity after 1 hour at 60 **°**C.

As for the 8 variants predicted by the Rosetta ddg_monomer program, I189H showed the highest residual activity with an 8-fold improvement relative to WT (Fig. 4). However, this mutation also resulted in a 95% loss of specific activity relative to wild type, most likely because Ile189, which is in the same co-factor loop as His192, also interacts directly with the thiazolium ring of ThDP^47^. Although both the I189H and H192P mutations were located in loop8, the residual activity of I189H was almost 1.6-fold higher than that of H192P, indicating that I189H introduced a greater rigidification of loop8. This highlights both the powerful capability of Rosetta in predicting stabilising variants, but also a key challenge in that Rosetta would not discriminate against mutations that potentially impair function. By contrast, the consensus approach in such sensitive areas is more pragmatic as it selects from functional mutations that exist in natural variants of the enzyme, although it would tend to provide more modest stability enhancements such as those of H192P and A282P.

The most stable variant predicted by Rosetta was D143K but that variant only increased the residual activity by 50% relative to wild type (Fig. 4). The residual activity of T245N was the lowest among all purified variants although it had a predicted ΔΔ*G* value of −4.1. Combining these 8 variants with the 41 tested above, for statistical analysis, we found that the qualitative stability changes of 32 variants were predicted correctly by the Rosetta in 65.3% of cases. Correlating the residual activities of the purified TK variants (Fig. 4) with their ΔΔ*G* values (Supplementary Fig. S9A) shows that 9 of 15 variants were predicted correctly by Rosetta, with five stable variants and four unstable variants relative to wild type, giving a prediction accuracy of 60%.

Variants showing improved residual activities were assessed further for their impact on other measures of stability, namely their thermal transition mid-points, *T*_m_, half-lifes of inactivation, and activities at elevated temperature. The thermal transition mid-point, *T*_m_, of each variant was measured from intrinsic fluorescence using an Optim1000 (Figure S7A). The *T*_m_ of 69.9 °C for D143K was essentially unchanged from that of wild-type (70.4 °C), whereas I189H marginally increased the *T*_m_ to 72.3 °C. The consensus variants H192P, A282P and double variant H192P/A282P, each increased the *T*_m_ to 74.0, 74.9 and 75.0 °C respectively, which were around 4–5 °C higher than that of wild type (Table 2). The increased *T*_m_ values are therefore all consistent with the improved residual activities observed for these variants after incubation for 1 hour at 60 °C.

From the unfolding curves reported using the barycentric mean fluorescence (BCM), a second transition was observed for H192P and H192P/A282P, which was not previously seen for wild type or the other two variants A282P and D143K (Supplementary Fig. S7A). As the low temperature baseline, and early transition map onto the wild-type curve, it appears that the H192P amino acid substitution selectively stabilised a part of the TK structure, leading to a separate transition at higher temperatures. In the structure of TK, Trp196 is directly connected to loop8 185–192 by a small helix (Supplementary Fig. S8). Local stabilisation of the cofactor-binding loop by the H192P could therefore be reported directly by Trp196 as a new transition at elevated temperature. Interestingly, the MD simulations also showed that loop8 185–192 and the regions nearby underwent dramatic fluctuations at elevated temperature, consistent with the new transition being attributable to stabilisation of this co-factor loop.

The variant H192P/A282P showed the highest *T*_m_ value among all the variants tested, and this might be contributed to by both the H192P and A282P amino acid substitutions. The interactions added or removed by the mutations are investigated in the ‘MD simulation analysis of variants’ below. A new salt bridge was formed between Glu275 and Lys280 in the variant A282P, and new hydrogen bonds were formed in loop8 (185–192) of variant H192P compared to the wild type. All of these new interactions were detected in the double variant H192P/A282P, suggesting that the increased stability was due to the additive effects of H192P and A282P.

The *T*_m_-values of the other 13 purified variants were also investigated to test the performance of Rosetta against this measure of thermodynamic stability (Supplementary Fig. S9B). As found above for residual activity, no obvious correlation was observed between the predicted ΔΔ*G* and the *T*_m_-values for these variants. Six of 11 variants were in zone IV, and none in zone I, resulting in a prediction accuracy of 46.2%. However, more samples would be required for a robust statistical analysis of any such correlation between predicted ΔΔ*G* and experimental *T*_m_-values for TK.

The half-life for the loss of enzyme activity at 60 °C was also determined as a measure of kinetic stability, for the five TK variants that had been shown to have improved residual activities relative to WT (Fig. 4). All five variants had a lower degradation rate constant *k*_d_ than for wild type, indicating that the variants deactivated more slowly (Supplementary Fig. S7B). H192P/A282P improved the half-life 3-fold relative to wild type, whereas A282P, H192P and I189H had 1.5-fold, 2-fold and 2.5-fold improved half-lifes, respectively (Table 2).

Michaelis-Menten kinetics at saturating (50 mM) Li-HPA were carried out to better understand the influence of mutations upon the enzyme kinetics at 22 °C. All variants had a similar *K*_m_ to wild type, indicating that these mutations did not significantly affect the interactions of GA within the active site (Table 2). I189H showed lowest specific activity among all wild-type and mutant TKs, and this could be contributed to a 40-fold decrease in *k*_*cat*_ to 1.5 s^−1^. This reflects the disruption of hydrophobic interactions with the thiazolium ring of ThDP, as discussed above, due to this mutation. Interestingly, the *k*_cat_ of variant H192P/A282P was improved 1.3-fold relative to wild type, which was not the case for either H192P or A282P. The improved *k*_*cat*_ could have resulted from an improvement in the flexibility of another co-factor loop (loop21), detected in MD simulations, as this might facilitate binding of co-factor (Supplementary Fig. S10). By contrast, A282P lost around 50% of the specific activity found in wild type, which is reflected in a 1.4-fold lower *k*_*cat*_ relative to wild type. The *K*_m_ of D143K is the lowest of the five variants. However, the *k*_*cat*_ achieved was also lower than wild type, which resulted in a comparable *k*_cat_/*K*_m_ and specific activity to that of wild type.

The optimum temperature range for the wild-type *E. coli* TK enzyme activity has been reported as 20–40 °C^14^. To investigate whether the variants functioned well at higher temperatures, we tested the catalytic activity of wild-type and variant TKs at the particularly challenging temperatures of 60 °C and 65 °C (Supplementary Fig. S7C, D). At high temperature, HPA can degrade independently, but this was observed in control experiments to be less than 5% within 1 h at 65 °C. At 60 °C, both the wild type and variants achieved increased specific activity compared to that at 22 °C (Table 2). The variant, H192P/A282P showed the highest specific activity of 254.7 μmol mg^−1^min^−1^ at 60 °C, a 4.3-fold improvement relative to that at 22 °C, and 2.8-fold higher than that of the wild type at 60 °C. This indicates an improved potential of H192P/A282P to be used in bioconversions at elevated temperatures.

I189H, surprisingly, achieved a specific activity of 40.9 μmol mg^−1^min^−1^ at 60 °C which was 21.5-fold higher than that at 22 °C, whereas the specific activity of the wild type increased only 1.8-fold when the reaction temperature was shifted to 60 °C. Additionally, the wild type and all variants, except H192P/A282P, displayed lower final conversions of erythrulose at 60 °C, compared to the 50 mM maximum observed at 22 °C (Supplementary Fig. S7C). This suggests that the high temperature denatured the enzymes before the substrates could be fully converted, and is consistent with the enzyme half-lifes at 60 °C. At 65 °C, the variants and wild type all demonstrated a significant loss of activity during the reaction, as indicated by the even lower final conversions to erythrulose compared to that at 60 °C (Supplementary Fig. S7D). However, H192P/A282P achieved a 5-fold higher initial activity compared to wild type, resulting in a 4-fold greater final conversion to erythrulose. Recently, a TK from the thermophilic microorganism *Geobacillus stearothermophilus* was characterised to have an optimal temperature range of 60–70 °C, and retained 100% activity for 3 days at 65 °C, which is more stable than our double variant. This enzyme has been engineered to convert unnatural substrates including aliphatic aldehydes^48^, (2 S)-hydroxyaldehydes^49^ and arylated substrates^50^ in recent studies, implying the potential of thermostable TK in the improvement of catalytic properties by mutagenesis.

### MD simulation analysis of variants

**Figure 5:**
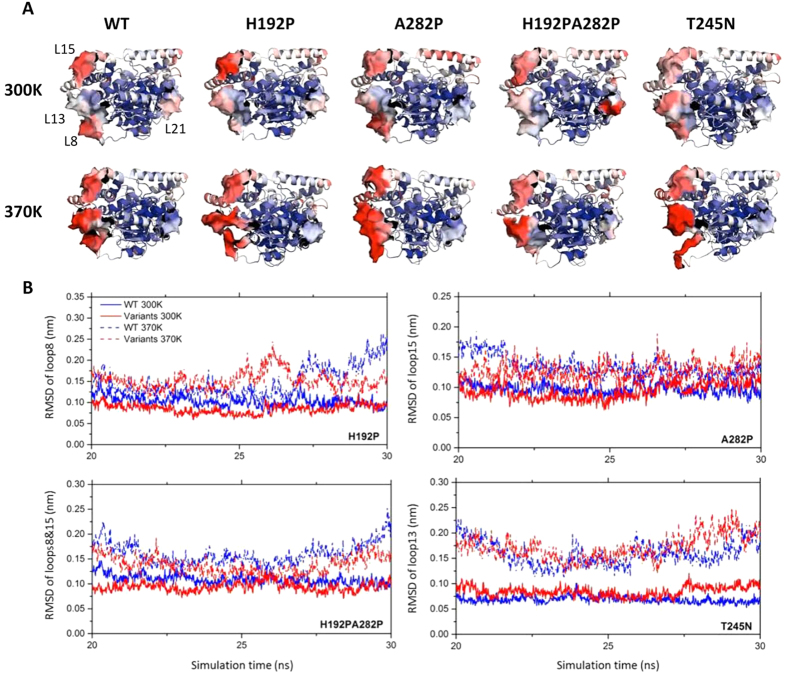
Flexibility of wild-type and mutant TKs. (**A**) Comparison of wild-type and mutant TKs structures coloured by normalized RMSF at 300 K and 370 K. Each structure was achieved from the average of the last 10-ns trajectory of one simulation. The surfaces of loops8, 13, 15, 21 are displayed and only those of WT at 300 K are labelled. (**B**) The RMSD of loops8, 13, 15 of wild-type and mutant TKs with the average conformations of last 10 ns as reference. For H192P/A282P, RMSD of loops8 and 15 were combined. Only the RMSD values of frames at 10-ps intervals were displayed for clarity and each value was the average of RMSD from triplicate simulations.

30-ns MD simulations were applied to examine the flexibility changes of variants H192P, A282P, H192P/A282P and T245N. The T245N variant was selected as it showed a similar specific activity but significantly decreased stability compared to WT (Fig. 4), thus it might be expected to display the opposite flexibility change to the three stabilising variants. The normalized RMSF values of each amino acid were calculated from the last 10 ns trajectory (Supplementary Fig. S10) and used for colouring structures of wild-type and mutant TKs (Fig. 5A). The local flexibilities of different variants were also examined by calculating the RMSD of the specific loops containing mutations in respect to its average conformation of last 10 ns (Fig. 5B).

At 300 K, the stable variants showed an increased local rigidity compared with the wild type in loop8 for H192P, loop15 for A282P, and both loop8 and15 for H192P/A282P, which all displayed lower RMSD or RMSF values than those of the wild type (Fig. 5). This is consistent with the new transition observed in variants containing H192P, as a result of localised stabilisation reported by W196 in loop8. Introduction of prolines at positions of His192 and Ala282 could have rigidified the local regions of TK and hence led to the improved thermostability. On the other hand, loop13 of variant T245N exhibited a significantly increased flexibility, suggesting that the mutation from Thr to Asn at position 245 might trigger a large conformational change around loop13.

Interestingly, the per-residue RMSF plots also showed the dynamic change of other regions beyond the five loops we identified (Supplementary Fig. S10A). For example, loop21 387–403 of variant H192P/A282P became more flexible than that of WT and other three variants. In order to confirm this, 100 frames were extracted from last 10 ns of one MD simulation at 300 K, and displayed in one picture (Supplementary Fig. 10B). Loop21 of H192P/A282P became more disordered compared to that of WT. Loop21 is a co-factor binding loop and interacts with loop8 of the second chain, across the dimer interface, to form one side of the active-site funnel with ThDP at the base (Supplementary Fig. S4). The increased flexibility of active sites has been observed frequently in thermostable proteins, and is believed to be linked to their higher temperatures of optimal activity^51^. Hence, the increased dynamics of loop21 could have contributed to the elevated *k*_cat_ of variant H192P/A282P (Table 2).

Loop8 and loop13 of WT are the two most thermally-sensitive loops and showed the greatest improvement in flexibility as the temperature increased from 300 K to 370 K (Fig. 1D). However, these two loops, along with loop15, were clearly rigidified at 370 K in the most thermostable variant, H192P/A282P, highlighting the critical role of flexible loops in regulating the protein thermostability (Fig. 5A). The variant H192P/A282P, therefore appears to have stabilized TK by rigidifying loops8, 13, 15, while increasing the enzyme activity by making loop21 more mobile. In agreement with the observation at 300 K, loop13 of variant T245N became more dynamic compared to that of wild type at 370 K. Also, loop8 of T245N apparently underwent an unfolding event at 370 K, which was not the case for WT and the other three variants, indicating its limited tolerance to high temperature (Fig. 5A). The local flexibilities of all three stable variants H192P, A282P, H192P/A282P were decreased, and that of T245N was increased compared to wild type, suggesting a good inverse correlation between flexibility and stability.

As a single mutation is unlikely to cause a significant change to the overall flexibility of a protein, especially one as large as TK, an appropriate method to examine the local flexibility around mutation sites is vital to predict the effect of mutations on protein stability. A method based on inspection of averaged structure from MD simulation trajectories has been used in a stability engineering strategy, FRESCO to analyse the flexibility effect of each single mutation^52^. Although the FRESCO strategy has proven useful^53^, the method used for predicting local flexibility is not straightforward since it is based on inspection instead of quantification. Additionally, the 100 ps MD simulations used in the FRESCO strategy would not be long enough for a protein to reach a stationary equilibrium phase. The method we used here took only conformations from the stationary phase into account, and an RMSD instead of RMSF calculation was used to allow detection of local region movement over the whole stationary phase, which provides an alternative approach for predicting local flexibility change caused by mutations, to the one used in the FRESCO strategy.

**Figure 6:**
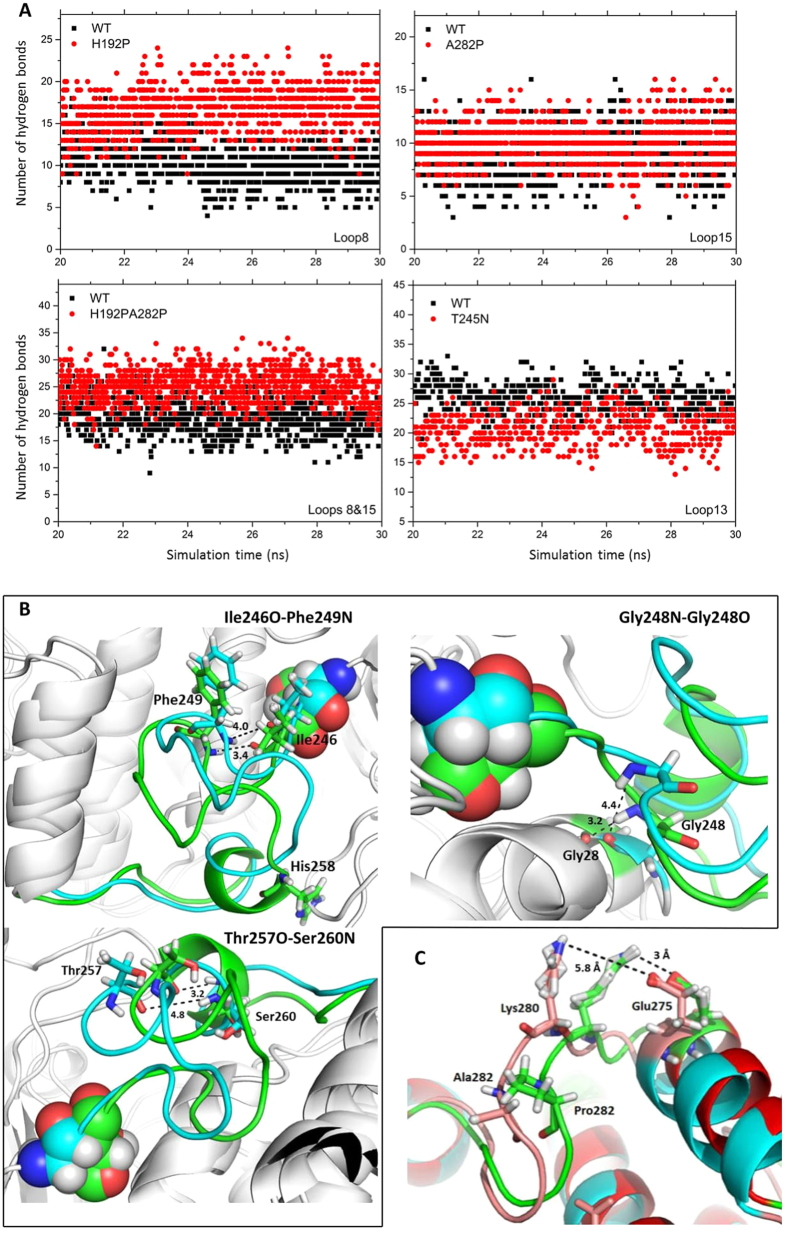
Hydrogen bonds and salt bridges formed by the loops of wild-type and mutant TKs. (**A**) Number of hydrogen bonds formed by loops (chain A and B) of wild type (black square) and variants (red circle) changed as the function of simulation time. Numbers of hydrogen bonds were calculated for last 10-ns simulation trajectories at 300 K and only those of frames at 10-ps intervals were displayed for clarity. (**B**) Ribbon diagrams showing the positions and distances of three hydrogen bonds found in WT (green) but not in variant T245N (cyan). The distance between atom O (red) and N (blue) in Å, was measured using *PyMol*. Residues, Thr245 of WT and Asn245 of T245N were displayed as spheres. WT and T245N structures were obtained from the average of the last 10 ns of simulation trajectories. (**C**) A ribbon diagram of loop8 showing positions and distances of the salt bridge in WT (pink) and A282P (green). WT and A282P structures were obtained from the average of the last 10 ns of simulation trajectories. Images were generated in *PyMOL*.

As shown in Supplementary Table S2, salt bridges and hydrogen bonds greatly influenced the flexibilities of the loops. It is useful to identify which interactions were removed or added by the mutations, and how these affect the flexibilities of the loops. The total number of hydrogen bonds involved in each loop kept changing during the simulation, and the average number of hydrogen bonds revealed differences for specific loops of WT and the variants (Fig. 6). Loop8 was rigidified by the H192P mutation, as a result of forming more hydrogen bonds on average in H192P (17) than for WT (11).

The variant T245N showed decreased thermostability and increased local flexibility compared to those of WT. Interestingly, the number of hydrogen bonds in loop13 decreased on average in T245N relative to those in WT (Fig. 6A). The mutation could have triggered a large conformational change and then removed several hydrogen bonds from loop13. In order to confirm that, we compared the structures of WT and T245N and identified three hydrogen bonds existing in WT but not in T245N (Fig. 6B). All of these hydrogen bonds were formed with the main chain atom N (blue) as a donor and the main chain atom O (red) as an acceptor. The distances between donors and acceptors (labelled on dashed lines) for WT (green) were smaller than the threshold of 3.9 Å, whereas those of T245N (cyan) exceeded the distance threshold for hydrogen bonds. Additionally, the small helix His258-His261, linked to loop13 (245–257) in WT, was denatured in the T245N variant, which would also contribute to the increased flexibility of the loop13 in T245N.

Loop15 of A282P showed decreased mobility compared to in wild type. Surprisingly, the average number of hydrogen bonds formed by the loop15 did not differ considerably between A282P and wild type (Fig. 6A). However, a salt bridge between Glu275 and Lys280 was formed in A282P but not in WT (Fig. 6C). In order to confirm this, the distance between the atom OE2 of Glu275 and the atom NZ of Lys280 was investigated during the last 10-ns simulation for the WT, A282P and H192P/A282P (Supplementary Fig. S11), revealing a shorter distance in A282P most of the time, and increasing occurrence of larger distances in H192P/A282P then WT. With the salt bridge distance threshold set to 3.2 Å, this salt bridge was intact for at a total of 3.1 ns, 5.1 ns, and 3.2 ns during the last 10 ns of simulations at 300 K for WT, A282P, and H192P/A282P, respectively. As shown in Fig. 6C, the A282P mutation could have restricted the mobility of its neighbour residue, Lys280 leading to the decreased distance between Glu275 and Lys280 in the variant A282P. This salt bridge survived a little longer in the variant H192P/A282P than in the WT, and the number of hydrogen bonds formed by loop8 and loop15 of H192P/A282P was significantly higher than those found in WT, which was in agreement with the fact that thermostable variant H192P/A282P showed increased local rigidity.

In this work, two strategies were applied to guide mutations in the flexible loops to engineer the thermostability of *E. coli* TK. According to the “back to consensus” concept, 40 single variants were designed in five flexible loops. A282P in loop15 was proven to be the most thermostable variant and its combination with H192P in loop8, a variant from our previous study, resulted in a double mutant H192P/A282P showing significantly improved thermostability and also catalytic activity compared to the wild type. In a second strategy, the Rosetta ddg_monomer program was used to predict the stability change of all possible 931 single variants within the same five target-loops. Eight variants with the lowest predicted ΔΔ*G* values were generated and characterised experimentally. Of these, the variant I189H showed an 8-fold increased kinetic stability relative to the wild type, but also led to a significant loss in activity. MD simulations of three stable variants H192P, A282P, H192P/A282P and one unstable variant T245N revealed a strong correlation between thermostability and rigidity, suggesting the important role of flexibility in engineering protein stability.

Based on a statistical analysis of the stability changes for all variants constructed, the qualitative prediction accuracy of the Rosetta ddg_monomer program reaches 65.3%. Two variants, A282P and R139C were identified from a library of 40 variants, with a success rate of 5%, guided by the “back to consensus” approach. Both of these variants were predicted accurately by Rosetta. However, when we ranked the ΔΔ*G* values of all 931 single variants, then A282P ranked only 72nd and R139C ranked 112th. These two variants would not be readily identified using Rosetta ΔΔ*G* values alone, as the library predicted by Rosetta is too large to be constructed using a site-directed mutagenesis approach. A flexibility prediction approach such as MD simulation could be used to identify variants with increased rigidity in the future to further reduce the library size.

Both of the two strategies investigated in this study were useful in guiding mutation candidates to flexible loops, and have the potential to be applied to other enzymes. Although the Rosetta ddg_monomer program had the higher success rate, as an alternative to pure computation design, the “back to consensus” strategy can be used for enzymes with no available structure. Rational design to engineer protein thermostability is still in progress and combination of different strategies could give an increased chance of success.

## Methods

All chemicals were obtained from Sigma-Aldrich, UK unless mentioned otherwise.

### Site-directed mutagenesis, overexpression and purification of enzymes

QuikChange XL Site-Directed Mutagenesis Kit (Agilent Technologies, US) was used to carry out site-directed mutagenesis with tktA gene in plasmid pQR791 as the template^54^. Wild-type and mutant TKs were expressed with an N-terminal His6-tag from *E. coli* XL10-Gold. A single colony was picked and transferred to 5 mL of LB Amp^+^ medium in a 50 mL falcon tube. Cultures were then transferred into 45 mL of LB Amp^+^ medium in a 250 mL shake flask, and incubated at 37 °C, 250 rpm, for an additional 8 hours. All proteins including wild-type and mutant TKs were purified with Ni-NTA spin columns (Qiagen, CA, USA), using the protocol provided. Purified protein was then transferred to Slide-A-Lyzer Dialysis Cassettes (Thermo Fisher Scientific, Paisley, UK) with 10 kDa molecular mass cut-off for dialysis against 2.4 mM thiamine diphosphate (ThDP), 9 mM MgCl_2_, 50 mM Tris-HCl pH 7.0 for 18 h at 4 °C. The final concentration of purified TK was measured using the Bradford method^55^, and OD_280_ measurements, independently.

### Microplate-based screening for thermostable variants

Microwell fermentation was carried out using 2 mL 96 deep-well square plates (DWP). Wells were filled with 900 μL LB Amp^+^ medium and inoculated with individual colonies of variants. DWP plates were sealed with breathable sterile film (VMR International, US) and incubated at 37 °C, 400 rpm for 18 h. Reaction plates were generated by transferring 200 μL cells to 96-well PCR plates and then centrifuged at 4000 rpm 30 minutes to collect cell pellets. Reaction plates were thawed from −80 °C resulting in freeze-thaw lysis of cells. The cells were resuspended in 50 μL 18 mM MgCl_2_, 4.8 mM ThDP, 50 mM Tris-HCl, pH 7.0 and inoculated for 30 minutes before heating at 60 °C for 1 h in a thermal cycler. Reactions were initiated by adding 50 μL 100 mM hydroxypyruvic acid (HPA), 100 mM glycolaldehyde (GA), 50 mM Tris-HCl, pH 7.0 at 22 °C, and then quenched after 60 min with 1 vol. 0.2% (V/V) trifluoroacetic acid (TFA). Samples were analysed by HPLC (Dionex, CA, USA) as previously^7^ to determine the concentration of L-erythrulose against a standard curve. For screening purposes only, an approximate specific activity of enzymes in lysates was estimated by dividing the initial activity of samples by the OD_600_ of culture.

### Temperature inactivation of holo-TK

Wild-type and mutant TKs were purified and then diluted to 0.1 mg/mL by dialysis buffer. The half-life of enzyme activity was measured in triplicate by placing 100 μL enzymes at 60 °C. Samples were removed at different times and then cooled to 25 °C. Reactions were initiated at 22 °C by adding 50 μL of 150 mM Li-HPA and 150 mM GA in 50 mM Tris-HCl, pH 7.0, then quenched at various times over 60 min by adding 10 μL sample into 190 μL 0.1% (v/v) TFA, prior to erythrulose determination by HPLC. A first-order deactivation rate constant (*k*_d_) was measured by linear regression of ln(residual activity) versus the incubation time (t). The half-life (t_1/2_) of each variant at 60 °C was calculated by Eq. (1).


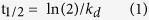


### TK activity measurement at high temperature

Purified wild-type and variants of *E. coli* TK were prepared at 0.1 mg/mL, with 2.4 mM TPP, 9 mM MgCl_2_ and 50 mM Tris-HCl, pH 7.0. Enzymes were then incubated for 5 min by placing 100 μL samples into a water bath equilibrated at 60 or 65 °C. Sample temperatures were monitored using a digital wired-thermometer (Topac, USA) and shown to equilibrate within 5 min. Reactions were initiated by addition of pre-warmed 50 μL of 150 mM Li-hydroxypyruvate (HPA), 150 mM glycolaldehyde (GA) in 50 mM Tris-HCl, pH 7.0. Aliquots of 10 μL were quenched at various times over 120 min with 190 μL of 0.1% (v/v) trifluoroacetic acid (TFA). Triplicate reactions were analysed by HPLC. Specific activities were determined as initial rate/enzyme concentration.

### Enzyme kinetics

Kinetic parameters were obtained at saturating 50 mM Li-HPA levels and a range of 4–80 mM GA in final conditions of 50 mM Tris-HCl, 2.4 mM ThDP, 9 mM MgCl_2_, pH 7.0. The mixtures containing enzymes (0.067 mg/mL) and substrates were incubated 22 °C for 2 h. Aliquots of 10 μL were quenched at various times by adding 190 μL of 0.1% (v/v) TFA. Triplicate reactions were monitored using HPLC as above. All data were fitted by non-linear regression to the Michaelis–Menten equation to determine the *K*_M_ and *k*_cat_ of wild-type TK and the variants using software *OriginPro9.0*.

### Thermal transition mid-point, *T*_m_, measurements

The *T*_m_-values of TK variants were measured in an Optim1000 (Unchained Laboratories, Wetherby, UK) via their intrinsic fluorescence. The microcuvette arrays were loaded with 9 μL of 1.0 mg/mL sample and excited with a 266 nm laser. The fluorescence was measured as a function of temperature in the range of 30–90 °C with steps of 1 °C, equilibration time of 30 s at each temperature, and a temperature tolerance of 0.5 °C. Barycentric mean fluorescence (λ_bcm_) was used as the analysis method for Optim1000 results, which was defined as Eq. (2).


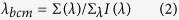


where λ is wavelength and I(λ) is the fluorescence intensity at wavelength. The λ _bcm_ indicates a change in the average wavelength of fluorescence emission, which was normalized for comparative purposes for different variants and fit to a two-state transition model to determine *T*_m_ as previously^56^.

### B-Factor analysis

The B-Factors of wild-type TK (PDB ID: 1QGD) were extracted from the pdb structure file using the B-FITTER software^33^. This tool calculates the amino acid B-Factor as an average of B-Factor of all the atoms of an amino acid in a given protein excluding hydrogen. Since TK is a homodimer, the B-Factor of each residue was calculated by averaging the B-factor values of the same residue from chain A and chain B.

### Atom depth calculations

The DEPTH server (http://mspc.bii.a-star.edu.sg/tankp/intro.html) was used to calculate atom depths for TK, defined as the distance of the atom from the nearest surface water molecule^57^. The default conditions used for calculating atom depth of TK were: number of solvating cycles, 25; solvent neighbourhood radius, 4.2 and minimum number of neighbourhood waters, 2.

### Salt bridges and hydrogen-bond analysis

Analysis of salt bridges was carried out with the visual molecular dynamics (VMD) program^58^. The distance threshold was set as 3.2 Å and PDB structure (1QGD) was used as the input. HBPLUS v.3.06 program was used to calculate the number of hydrogen bonds in the TK structure^59^ setting maximum distances for D-A and H-A bonds at 3.9 Å and 2.5 Å respectively [D refers to the donor atom; A, the acceptor; H, the hydrogen atom].

### Molecular dynamics simulations

Molecular dynamics simulation software Gromacs v 5.0 was used to investigate the structural flexibility of wild-type TK (PDB ID 1QGD) and variants constructed with the PyMol Mutagenesis Wizard (Schrödinger, USA). Simulations were carried out using the OPLS-AA force field. The initial structure was solvated in a cubic simulation box with a layer of water at least 10.0 Å from the protein surface. Sufficient Na^+^ was added to neutralize the negative charges in the system. The whole system was minimized using the steepest descent method (2000 steps) plus the conjugate gradient method (5000 steps). Two 50 ps position-restricted simulations were performed under NVT and NPT ensembles respectively with heavy atoms and C_α_-atoms fixed. Finally, a 30 ns MD simulation was performed in triplicate on the whole system at 300 K, 330 K and 370 K. All bond lengths were constrained using the LINCS algorithm and the time step of simulation was set to 2 fs. Trajectories were saved at every 2 ps and post-analysis was performed using standard Gromacs tools. RMSDs (root-mean-square deviations) were calculated using the starting structure of each simulation as a reference.

### ΔΔ*G* calculations and mutational scanning

The relative change in folding free energy due to point mutations, ΔΔG, was predicted for residues in flexible regions using the Rosetta ddg_monomer application^38^. Here the TK variant G540Stop with a truncated C-terminal domain, instead of the full-length wild-type structure, was used as the input for Rosetta due to its limitation on computational resource. G540stop has been reported to have an increased catalytic rate compared to WT TK, whereas the C-terminal domain has an as yet unknown function, and is not thought to contribute significantly to the stability of TK^47^. For the Rosetta ddg_monomer program, we used the high-resolution algorithm which allows a small degree of backbone conformational freedom. Based on the high resolution protocol, the flags used for the ddg_monomer executable were as follows –in:files target.pdb –ddg::mut file mutation.mutifle -ddg::weight_file soft_rep_design –fa_max_dis 9.0 -ddg::iterations 50 -ddg::min_cst false -ddg:: mean true -ddg::min false -ddg::sc_min_only false -ddg:: ramp_repulsive true).

## Additional Information

**How to cite this article**: Yu, H. *et al*. Two strategies to engineer flexible loops for improved enzyme thermostability. *Sci. Rep.* **7**, 41212; doi: 10.1038/srep41212 (2017).

**Publisher's note:** Springer Nature remains neutral with regard to jurisdictional claims in published maps and institutional affiliations.

## Supplementary Material

Supplementary Information

## Figures and Tables

**Table 1:** Design of variants based on β-turn amino acid positional preference.

Loops	β-turns	Type	Possible mutations
Loop6 138–148	139RPGH142	II	R**139**C,P H**142**C,D,K,Q,S,T
Loop13 245–257	246IIGF249	II	I**246**C,P I**247**A,E,K,P F**249**C,D,K,Q,S,T
	251SPNK254	I	K**254**C,G,N,D,S,T,P
	252PNKA255	I	
	254KAGT257	II	
Loop15 278–287	282APFE285	II	A**282**C,P F**284**G,N E**285**C,D,K,Q,S,T
Loop17 331–337	334PSDF337	I	F**337**C,G,N

**Table 2:** Characteristics of wild-type and mutant TKs.

	Specific activity 22 °C	(μmol mg^−1^min^−1^) 60 °C	*T*_m_ (°C)	Half life t_1/2_ (min)	*K*_m_ (mM)	*k*_cat_ (s^−1^)	*k*_cat_/*K*_m_ (s^−1^ M^−1^)
WT	48.8 (0.4)	89.8 (1.4)	70.4 (0.3)	22.9	20.5 (1.3)	62.3 (2.4)	3039
D143K	46.7 (1.6)	—	69.9 (0.4)	25.2	16.1 (1.2)	48.1 (3.2)	2988
I189H	1.9 (0.1)	40.9 (1.1)	72.3 (0.2)	56.8	18.0 (0.8)	1.5 (0.03)	833
H192P	57.3 (1.0)	158.0 (3.7)	74.0 (0.2)	43.9	18.0 (1.2)	65.5 (1.9)	3639
A282P	22.3 (0.5)	89.3 (1.1)	74.9 (0.2)	36.1	17.6 (2.1)	45.5 (1.5)	2585
H192P/A282P	59.9 (1.4)	254.7 (2.3)	75.0 (0.1)	63.0	23.3 (1.4)	81.2 (4.2)	3485

## References

[b1] Sprenger G. A., Schorken U., Sprenger G. & Sahm H. Transketolase A of Escherichia coli K12. Purification and properties of the enzyme from recombinant strains. European journal of biochemistry/FEBS 230, 525–532 (1995). 10.1111/j.1432-1033.1995.0525h.x 7607225

[b2] Draths K. M. . Biocatalytic Synthesis of Aromatics from D-Glucose - the Role of Transketolase. Journal of the American Chemical Society 114, 3956–3962 (1992).

[b3] Demuynck C., Bolte J., Hecquet L. & Dalmas V. Enzyme-Catalyzed Synthesis of Carbohydrates - Synthetic Potential of Transketolase. Tetrahedron Lett 32, 5085–5088 (1991).

[b4] Morris K. G. . Transketolase from Escherichia coli: A practical procedure for using the biocatalyst for asymmetric carbon-carbon bond synthesis. Tetrahedron-Asymmetr 7, 2185–2188 (1996).

[b5] Sprenger G. A. & Pohl M. Synthetic potential of thiamin diphosphate-dependent enzymes. J Mol Catal B-Enzym 6, 145–159 (1999).

[b6] Smith M. E. B., Hibbert E. G., Jones A. B., Dalby P. A. & Hailes H. C. Enhancing and Reversing the Stereoselectivity of Escherichia coli Transketolase via Single-Point Mutations. Adv Synth Catal 350, 2631–2638 (2008).

[b7] Hibbert E. G. . Directed evolution of transketolase activity on non-phosphorylated substrates. Journal of biotechnology 131, 425–432 (2007). 17825449 10.1016/j.jbiotec.2007.07.949

[b8] Hibbert E. G. . Directed evolution of transketolase substrate specificity towards an aliphatic aldehyde. Journal of biotechnology 134, 240–245 (2008). 18342970 10.1016/j.jbiotec.2008.01.018

[b9] Galman J. L. . Alpha,alpha’-Dihydroxyketone formation using aromatic and heteroaromatic aldehydes with evolved transketolase enzymes. Chemical communications 46, 7608–7610 (2010). 20835425 10.1039/c0cc02911d

[b10] Cazares A. . Non-alpha-hydroxylated aldehydes with evolved transketolase enzymes. Organic & biomolecular chemistry 8, 1301–1309 (2010). 20204200 10.1039/b924144b

[b11] Subrizi F. . Transketolase catalysed upgrading ofl-arabinose: the one-step stereoselective synthesis ofl-gluco-heptulose. Green Chem. 18, 3158–3165 (2016).

[b12] Payongsri P. . Rational substrate and enzyme engineering of transketolase for aromatics. Organic & biomolecular chemistry 10, 9021–9029 (2012). 23079923 10.1039/c2ob25751c

[b13] Payongsri P., Steadman D., Hailes H. C. & Dalby P. A. Second generation engineering of transketolase for polar aromatic aldehyde substrates. Enzyme Microb Technol 71, 45–52 (2015). 25765309 10.1016/j.enzmictec.2015.01.008

[b14] Jahromi R. R., Morris P., Martinez-Torres R. J. & Dalby P. A. Structural stability of E. coli transketolase to temperature and pH denaturation. Journal of biotechnology 155, 209–216 (2011). 21723889 10.1016/j.jbiotec.2011.06.023

[b15] Morris P., Rios-Solis L., Garcia-Arrazola R., Lye G. J. & Dalby P. A. Impact of cofactor-binding loop mutations on thermotolerance and activity of *E. coli* transketolase. Enzyme Microb Technol 89, 85–91 (2016). 27233131 10.1016/j.enzmictec.2016.04.003

[b16] Bommarius A. S. & Paye M. F. Stabilizing biocatalysts. Chemical Society reviews 42, 6534–6565 (2013). 23807146 10.1039/c3cs60137d

[b17] Dalby P. A. Strategy and success for the directed evolution of enzymes. Current opinion in structural biology 21, 473–480 (2011). 21684150 10.1016/j.sbi.2011.05.003

[b18] Goldsmith M. & Tawfik D. S. Enzyme engineering by targeted libraries. Methods in enzymology 523, 257–283 (2013). 23422434 10.1016/B978-0-12-394292-0.00012-6

[b19] Nestl B. M. & Hauer B. Engineering of Flexible Loops in Enzymes. ACS Catalysis 4, 3201–3211 (2014).

[b20] Furnham N. . Exploring the evolution of novel enzyme functions within structurally defined protein superfamilies. PLoS computational biology 8, e1002403 (2012). 22396634 10.1371/journal.pcbi.1002403 PMC3291543

[b21] Malabanan M. M., Amyes T. L. & Richard J. P. A role for flexible loops in enzyme catalysis. Current opinion in structural biology 20, 702–710 (2010). 20951028 10.1016/j.sbi.2010.09.005 PMC2994964

[b22] Gunasekaran K., Ma B. & Nussinov R. Triggering loops and enzyme function: identification of loops that trigger and modulate movements. Journal of molecular biology 332, 143–159 (2003). 12946353 10.1016/s0022-2836(03)00893-3

[b23] Damnjanovic J., Nakano H. & Iwasaki Y. Deletion of a dynamic surface loop improves stability and changes kinetic behavior of phosphatidylinositol-synthesizing Streptomyces phospholipase D. Biotechnology and bioengineering 111, 674–682 (2014). 24222582 10.1002/bit.25149

[b24] Herbert C. . Molecular mechanism of SSR128129E, an extracellularly acting, small-molecule, allosteric inhibitor of FGF receptor signaling. Cancer cell 23, 489–501 (2013). 23597563 10.1016/j.ccr.2013.02.018

[b25] Yedavalli P. & Rao N. M. Engineering the loops in a lipase for stability in DMSO. Protein engineering, design & selection: PEDS 26, 317–324 (2013). 10.1093/protein/gzt002 23404771

[b26] Yu H., Zhao Y., Guo C., Gan Y. & Huang H. The role of proline substitutions within flexible regions on thermostability of luciferase. Biochimica et biophysica acta 1854, 65–72 (2015). 25448017 10.1016/j.bbapap.2014.10.017

[b27] Wintrode P. L., Zhang D., Vaidehi N., Arnold F. H. & Goddard W. A. 3rd. Protein dynamics in a family of laboratory evolved thermophilic enzymes. Journal of molecular biology 327, 745–757 (2003). 12634066 10.1016/s0022-2836(03)00147-5

[b28] Mamonova T. B., Glyakina A. V., Galzitskaya O. V. & Kurnikova M. G. Stability and rigidity/flexibility-two sides of the same coin? Biochimica et biophysica acta 1834, 854–866 (2013). 23416444 10.1016/j.bbapap.2013.02.011

[b29] Paul M., Hazra M., Barman A. & Hazra S. Comparative molecular dynamics simulation studies for determining factors contributing to the thermostability of chemotaxis protein “CheY”. Journal of biomolecular structure & dynamics 32, 928–949 (2014). 23796004 10.1080/07391102.2013.799438

[b30] McClelland L. J. & Bowler B. E. Lower Protein Stability Does Not Necessarily Increase Local Dynamics. Biochemistry 55, 2681–2693 (2016). 27104373 10.1021/acs.biochem.5b01060

[b31] Yu H. & Huang H. Engineering proteins for thermostability through rigidifying flexible sites. Biotechnology advances 32, 308–315 (2014). 24211474 10.1016/j.biotechadv.2013.10.012

[b32] Parthasarathy S. & Murthy M. R. Protein thermal stability: insights from atomic displacement parameters (B values). Protein engineering 13, 9–13 (2000). 10679524 10.1093/protein/13.1.9

[b33] Reetz M. T., Carballeira J. D. & Vogel A. Iterative saturation mutagenesis on the basis of B factors as a strategy for increasing protein thermostability. Angewandte Chemie 45, 7745–7751 (2006). 17075931 10.1002/anie.200602795

[b34] Kim H. S., Le Q. A. T. & Kim Y. H. Development of thermostable lipase B from Candida antarctica (CalB) through in silico design employing B-factor and RosettaDesign. Enzyme and Microbial Technology 47, 1–5 (2010).

[b35] Le Q. A., Joo J. C., Yoo Y. J. & Kim Y. H. Development of thermostable Candida antarctica lipase B through novel in silico design of disulfide bridge. Biotechnology and bioengineering 109, 867–876 (2012). 22095554 10.1002/bit.24371

[b36] Fei B. . A multi-factors rational design strategy for enhancing the thermostability of Escherichia coli AppA phytase. Journal of industrial microbiology & biotechnology 40, 457–464 (2013). 23494709 10.1007/s10295-013-1260-z

[b37] Bommarius A. S., Blum J. K. & Abrahamson M. J. Status of protein engineering for biocatalysts: how to design an industrially useful biocatalyst. Current opinion in chemical biology 15, 194–200 (2011). 21115265 10.1016/j.cbpa.2010.11.011

[b38] Kellogg E. H., Leaver-Fay A. & Baker D. Role of conformational sampling in computing mutation-induced changes in protein structure and stability. Proteins 79, 830–838 (2011). 21287615 10.1002/prot.22921 PMC3760476

[b39] Morris P., Rios-Solis L., García-Arrazola R., Lye G. J. & Dalby P. A. Impact of cofactor-binding loop mutations on thermotolerance and activity of E. coli transketolase. Enzyme and Microbial Technology 89, 85–91 (2016). 27233131 10.1016/j.enzmictec.2016.04.003

[b40] Nikkola M., Lindqvist Y. & Schneider G. Refined structure of transketolase from Saccharomyces cerevisiae at 2.0 A resolution. Journal of molecular biology 238, 387–404 (1994). 8176731 10.1006/jmbi.1994.1299

[b41] Wen S., Tan T. & Zhao H. Improving the thermostability of lipase Lip2 from Yarrowia lipolytica. Journal of biotechnology 164, 248–253 (2012). 22982168 10.1016/j.jbiotec.2012.08.023

[b42] Diao H., Zhang C., Wang S., Lu F. & Lu Z. Enhanced Thermostability of Lipoxygenase from Anabaena sp. PCC 7120 by Site-Directed Mutagenesis Based on Computer-Aided Rational Design. Applied biochemistry and biotechnology (2015). 10.1007/s12010-015-1950-2 26686337

[b43] Jochens H., Aerts D. & Bornscheuer U. T. Thermostabilization of an esterase by alignment-guided focussed directed evolution. Protein engineering, design & selection: PEDS 23, 903–909 (2010). 10.1093/protein/gzq071 20947674

[b44] Guruprasad K. & Rajkumar S. Beta-and gamma-turns in proteins revisited: a new set of amino acid turn-type dependent positional preferences and potentials. Journal of biosciences 25, 143–156 (2000). 10878855

[b45] Hutchinson E. G. & Thornton J. M. A revised set of potentials for beta-turn formation in proteins. Protein science: a publication of the Protein Society 3, 2207–2216 (1994). 7756980 10.1002/pro.5560031206 PMC2142776

[b46] Thiltgen G. & Goldstein R. A. Assessing predictors of changes in protein stability upon mutation using self-consistency. PloS one 7, e46084 (2012). 23144695 10.1371/journal.pone.0046084 PMC3483175

[b47] Costelloe S. J., Ward J. M. & Dalby P. A. Evolutionary analysis of the TPP-dependent enzyme family. Journal of molecular evolution 66, 36–49 (2008). 18043855 10.1007/s00239-007-9056-2

[b48] Yi D. . A thermostable transketolase evolved for aliphatic aldehyde acceptors. Chemical communications 51, 480–483 (2015). 25415647 10.1039/c4cc08436e

[b49] Zabar J. A. . Engineering a Thermostable Transketolase for Unnatural Conversion of (2S)-Hydroxyaldehydes. Adv Synth Catal 357, 1715–1720 (2015).

[b50] Saravanan T. . Engineering a thermostable transketolase for arylated substrates. Green Chem. (2017).

[b51] Singh B., Bulusu G. & Mitra A. Understanding the thermostability and activity of Bacillus subtilis lipase mutants: insights from molecular dynamics simulations. The journal of physical chemistry. B 119, 392–409 (2015). 25495458 10.1021/jp5079554

[b52] Wijma H. J. . Computationally designed libraries for rapid enzyme stabilization. Protein engineering, design & selection: PEDS 27, 49–58 (2014). 10.1093/protein/gzt061 PMC3893934 24402331

[b53] Wu B. . Versatile Peptide C-Terminal Functionalization via a Computationally Engineered Peptide Amidase. ACS Catalysis 6, 5405–5414 (2016).

[b54] Martinez-Torres R. J., Aucamp J. P., George R. & Dalby P. A. Structural stability of *E. coli* transketolase to urea denaturation. Enzyme and Microbial Technology 41, 653–662 (2007).

[b55] Bradford M. M. A rapid and sensitive method for the quantitation of microgram quantities of protein utilizing the principle of protein-dye binding. Analytical biochemistry 72, 248–254 (1976). 942051 10.1016/0003-2697(76)90527-3

[b56] Chakroun N., Hilton D., Ahmad S. S., Platt G. W. & Dalby P. A. Mapping the Aggregation Kinetics of a Therapeutic Antibody Fragment. Molecular pharmaceutics 13, 307–319 (2016). 26692229 10.1021/acs.molpharmaceut.5b00387

[b57] Chakravarty S. & Varadarajan R. Residue depth: a novel parameter for the analysis of protein structure and stability. Structure 7, 723–732 (1999). 10425675 10.1016/s0969-2126(99)80097-5

[b58] Humphrey W., Dalke A. & Schulten K. VMD: visual molecular dynamics. Journal of molecular graphics 14, 33–38, 27–38 (1996). 8744570 10.1016/0263-7855(96)00018-5

[b59] McDonald I. K. & Thornton J. M. Satisfying hydrogen bonding potential in proteins. Journal of molecular biology 238, 777–793 (1994). 8182748 10.1006/jmbi.1994.1334

