# Supplementary material for: Two strategies to engineer flexible loops for improved enzyme thermostability
Source: Sci Rep. 2017 Feb 1;7:41212. doi: 10.1038/srep41212 (PMC5286519; doi:10.1038/srep41212)
Supplement: Supplementary Information [file srep41212-s1.pdf]

Supplementary Information for

Two strategies to engineer flexible loops for improved enzyme thermostability

Haoran Yu, Yihan Yan, Cheng Zhang, Paul A. Dalby*

Department of Biochemical Engineering, University College London, Gordon Street, London, WC1H 0AH, United Kingdom

*Correspondence and requests for materials should be addressed to P.A.D. (email: p.dalby@ucl.ac.uk)

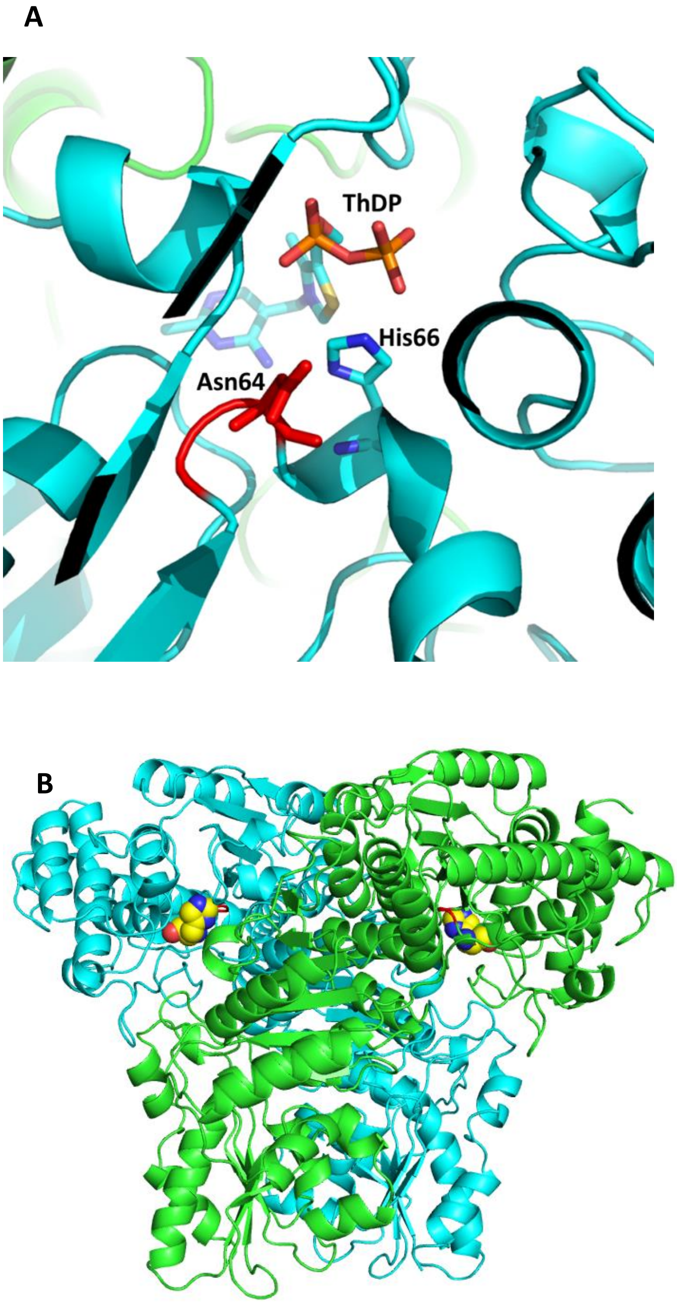

Supplementary Figure S1. The position of loop 3 in TK. (A), loop3 located near to cofactor and His66. (B), loop3 located close to the interface of two chains. Loop 3 is coloured by red, while His66 is displayed as spheres. The images were generated by *PyMol* with the PDB structure, 1QGD as the input.

Supplementary Table S1. Flexibility rank of loops based on B-factors

Rank	Loops	B-Factor	Depth (Å)	Rank	Loops	B-Factor	Depth (Å)
**1**	L15 278-287	29.90	4.42	**21**	L38 631-640	15.83	5.33
**2**	L17 331-337	25.63	4.37	**22**	L11 214-220	15.67	4.49
**3**	L13 245-257	23.64	4.98	**23**	L25 451-454	14.81	5.21
**4**	L14 261-266	22.93	4.67	**24**	L31 519-529	14.01	4.99
**5**	L33 545-551	22.56	3.93	**25**	L19 372-376	13.83	7.31
**6**	L1 23-27	19.80	6.11	**26**	L2 44-58	13.67	5.51
**7**	L16 295-296	19.41	4.64	**27**	L3 63-64	13.29	11.67
**8**	L30 509-513	19.08	5.60	**28**	L7 173-177	13.12	5.27
**9**	L36 601-607	18.95	4.47	**29**	L9 196-200	12.99	4.96
**10**	L39 648-651	18.76	4.61	**30**	L20 380-382	11.93	7.84
**11**	L37 624-627	18.50	4.69	**31**	L27 467-477	11.10	8.53
**12**	L32 537-538	18.15	4.26	**32**	L23 425-428	10.89	6.95
**13**	L4 79-83	18.13	4.87	**33**	L10 209-210	10.79	6.06
**14**	L12 234-238	17.82	4.99	**34**	L26 461-463	10.71	9.05
**15**	L18 351-356	17.68	4.10	**35**	L28 484-488	10.61	5.67
**16**	L21 387-403	17.44	4.74	**36**	L24 440-441	9.79	14.33
**17**	L6 138-148	17.26	5.02	**37**	L29 492-495	9.60	10.79
**18**	L8 185-192	16.97	5.10	**38**	L22 407-410	9.52	10.92
**19**	L35 592-593	16.79	4.03	**39**	L34 583-585	9.20	10.27
**20**	L5 90-117	16.44	7.17				

Supplementary Table S2. Interactions detected in the five surface loops

	Rank	Salt Bridges	Hydrogen Bonds [a]	
Loop11 214-220	22	Asp215-Lys244	1. Arg214-Glu206	2. Arg214-Asp200
		Glu206-Arg214	3. Asp215-Lys244	4. Ile216-Met242
		His219-Asp35	5. Ile216-Lys244	6. Asp217-His219
			7. Gly218-Lys244	8. His219-Ile246
			9. His219-Asp35	10. Asp220-Ala222
			11. Asp220-Ser223	12. Asp220-Ile224
Loop15 278-287	1	None	1. Gly278-Arg274	2. Trp279-Arg274
			3. Trp279-Lys280	4. Lys280-Arg274
			5. Try281-Arg274	6. Ala282-Glu285
			7. Phe284-Asp17	8. Pro287-Ile290
			9. Pro287-Tyr291	
Loop17 331-337	2	None	1. Gly331-Arg327	2. Gly331-Gln510
			3. Gly331-Arg454	4. Glu332-Arg327
			5. Glu332-Lys330	6. Pro334-Phe337
			7. Asp336-Lys340	8. Phe337-Aln341
Loop33 545-551	5	Asp545-Arg579	1. Asp545-Arg579	2. Asp545-Tyr568
			3. Ala547-Lys577	4. Glu551-Lys577
Loop35 592-593	19	None	1. Gln592-Ala588	2. Gln592-Phe589
			3. Gln592-Arg597	4. Gln592-Arg538
			5. Asp593-Ala595	6. Asp593-Tyr596
			7. Asp593-Arg597	

[a] For the hydrogen bonds appearing between two residuals more than once, only one time was count. For instance, there are two hydrogen bonds detected between side chains of Arg214 and Glu206, 214Arg NH1-206Glu OE2 and 214 Arg NH2- 206Glu OD1. In this case, only Arg214-Glu206 was listed in the table.

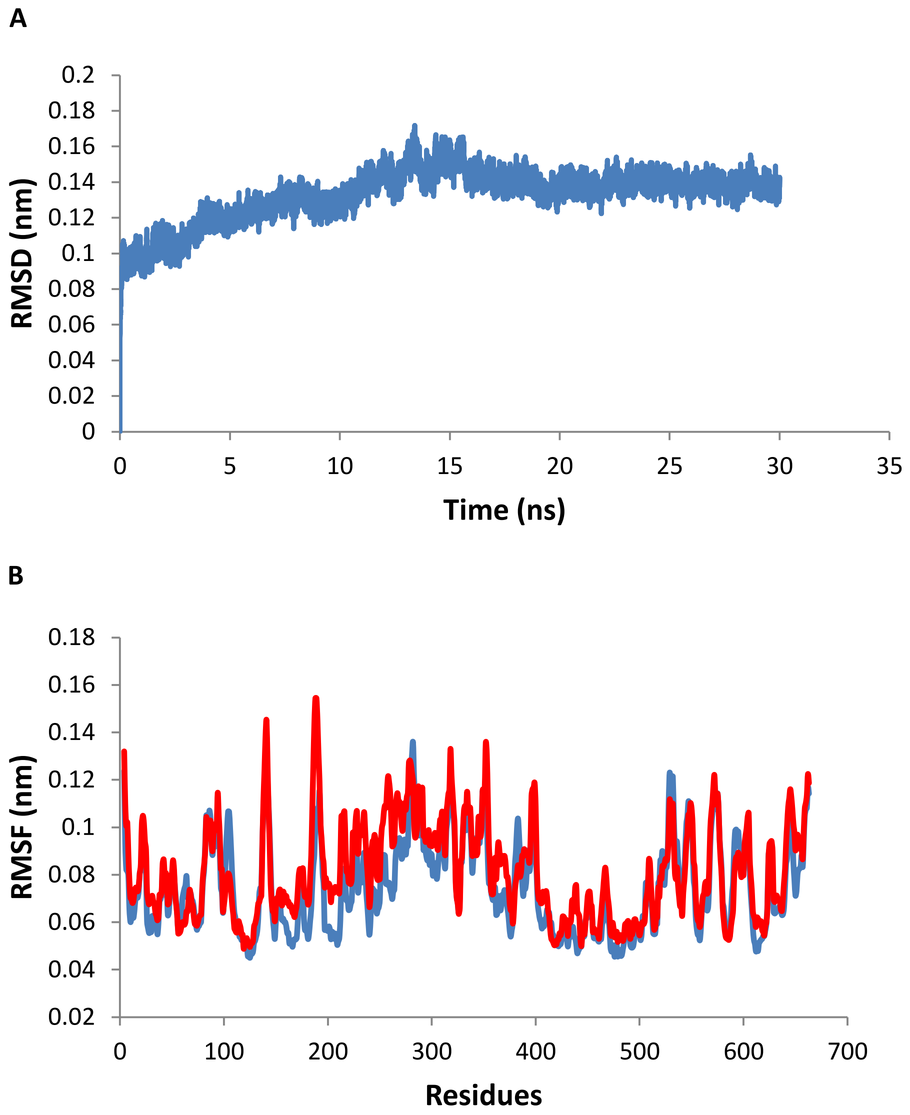

Supplementary Figure S2. RMSD and RMSF values of wild-type TK. (A), Backbone RMSD versus simulation time. (B), RMSF values calculated from two independent MD simulation runs at 300 K.

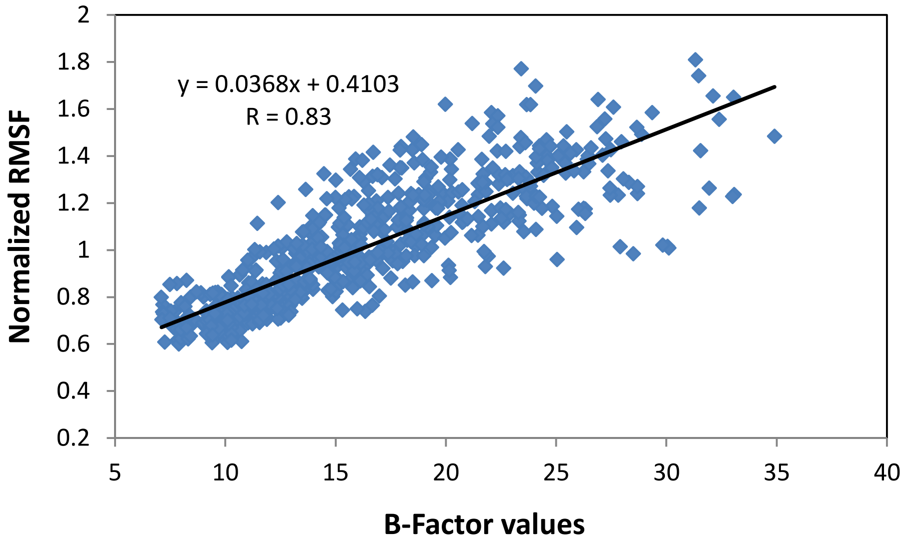

Supplementary Figure S3. Correlation between normalized RMSF and B-Factor values.

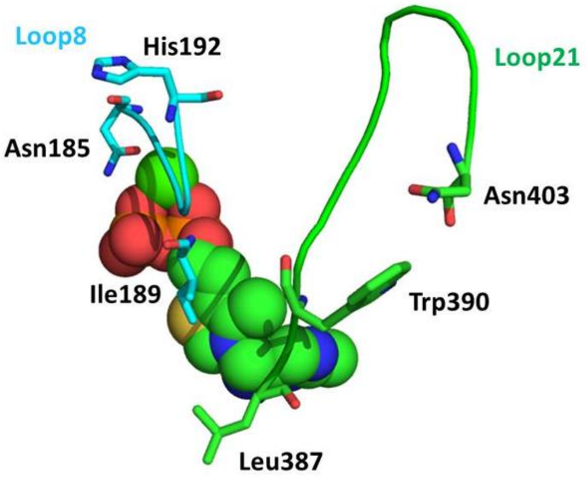

Supplementary Figure S4. Relative positions of loop8 (cyan) and loop21 (green). The ThDP cofactors and Ca^{2+} ion are shown as spheres. Important residues are represented as sticks. The image was generated by *PyMol*

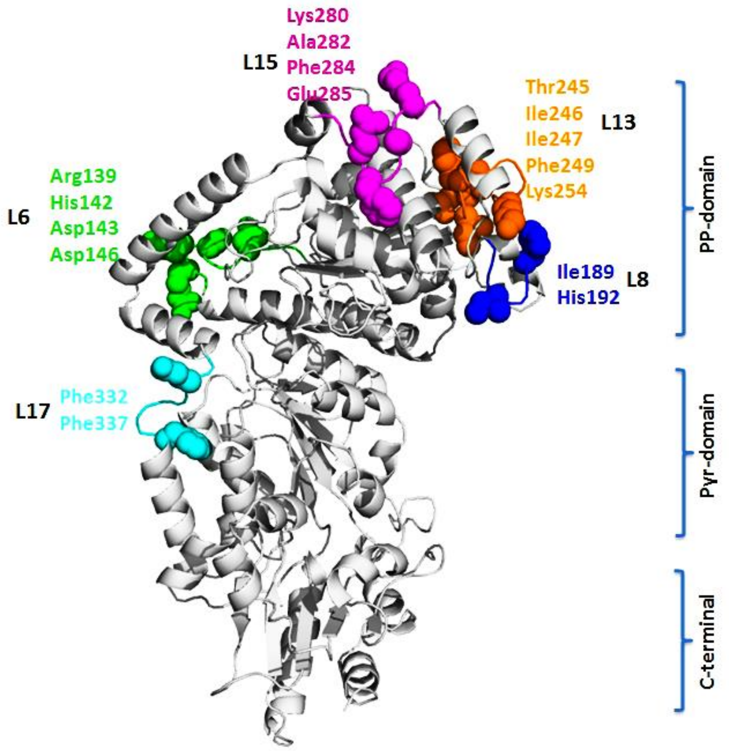

Supplementary Figure S5. Locations of the flexible loops and mutations sites. Only one chain of the TK homodimer is shown. Five targeted loops are coloured differently. The mutation sites are displayed as spheres. Image was generated in *PyMOL*

Supplementary Table S3. Designations of TK β-Turns

No.	β-Turn	Type	No.	β-Turn	Type	No.	β-Turn	Type
1	48NPQN51	I	17	188SIDG191	I	33	397NEDA400	VIII
2	49PQNP52	VIII	18	217DGHD220	I	34	399DAAG402	I
3	51NPSW54	I	19	234VTDK237	I	35	407YGVR410	IV
4	54WADR57	I	20	246IIGF249	II	36	439EYAR442	I
5	62LSNG65	IV	21	248GFGS251	IV	37	467GEDG470	I
6	89NFRQ92	II	22	251SPNK254	I	38	470GPTH473	I
7	92QLHS95	II	23	252PNKA255	I	39	471PTHQ474	I
8	95SKTP98	IV	24	254KAGT257	II	40	475PVEQ478	IV
9	98PGHP101	VIII	25	260SHGA263	IV	41	485TPNM488	II
10	102EVGK105	II	26	282APFE285	II	42	509RQDG512	I
11	103VGKT106	IV	27	334PSDF337	I	43	542VLKD545	VIII
12	106TAGV109	II	28	372LPEF375	I	44	550PELI553	VIII
13	115PLGQ118	II	29	379SADL382	IV	45	583MPST586	VIII
14	139RPGH142	II	30	390WSGS393	I	46	602PKAV605	I
15	143DIVD146	VIII	31	395AINE398	I	47	624GLNG627	I
16	175LGKL178	I	32	396INED399	I			

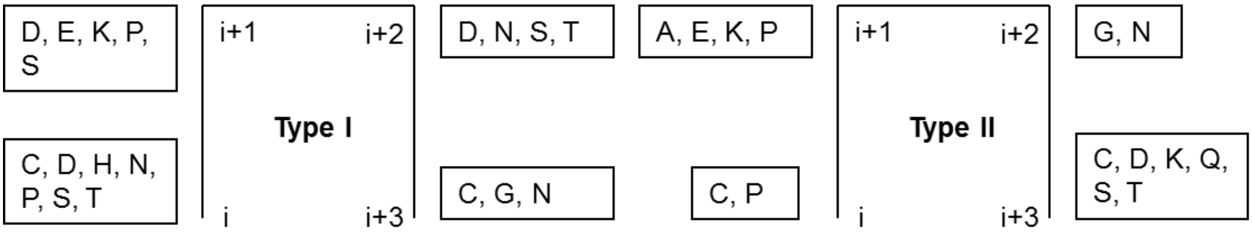

Supplementary Figure S6. Amino acid positional preference for type I and type II β-Turn. Determined by Guruprasad and Rajkumar[1]

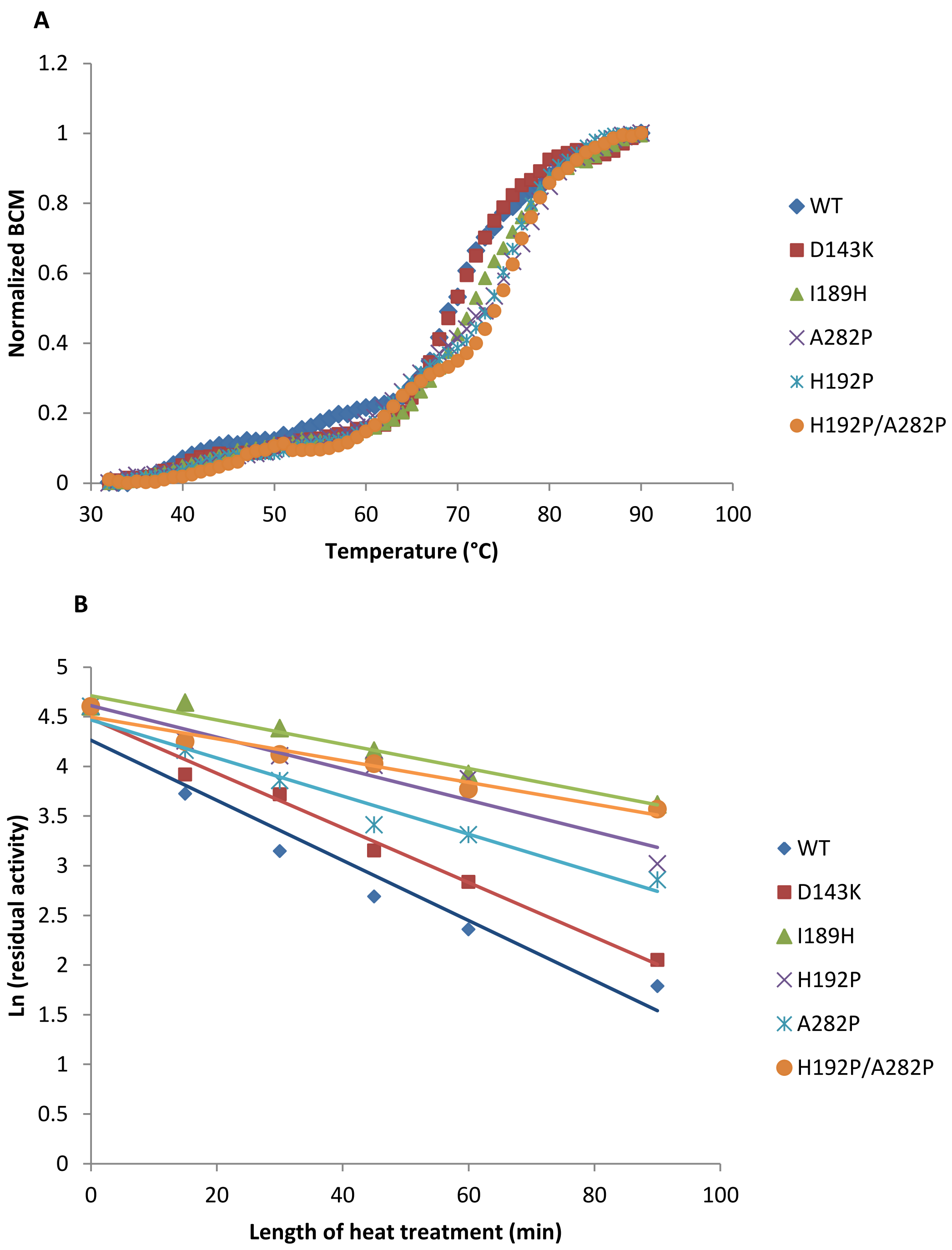
A
1.2
1
0.8
0.6
0.4
0.2
0
Normalized BCM
30
40
50
60
70
80
90
100
Temperature (°C)
WT
D143K
I189H
A282P
H192P
H192P/A282P
B
5
4.5
4
3.5
3
2.5
2
1.5
1
0.5
0
Ln (residual activity)
0
20
40
60
80
100
Length of heat treatment (min)
WT
D143K
I189H
H192P
A282P
H192P/A282P

C

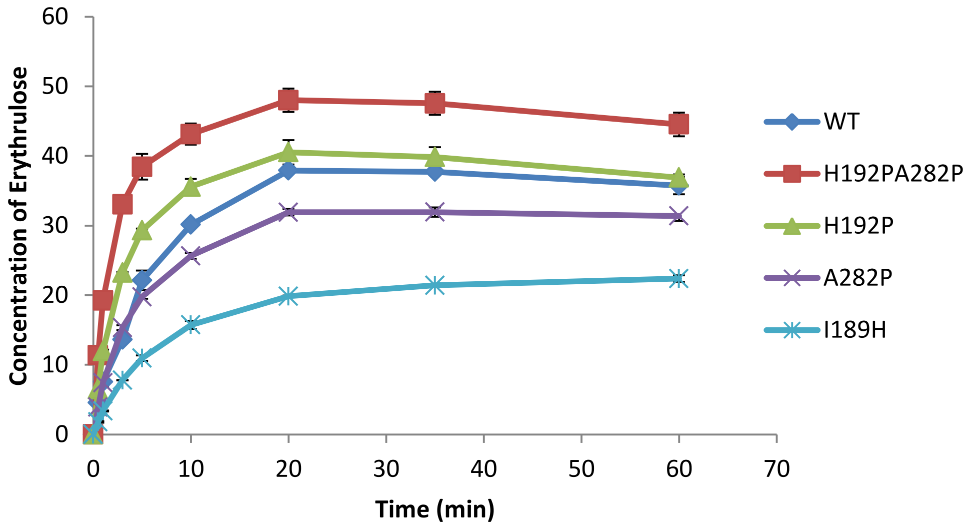

D

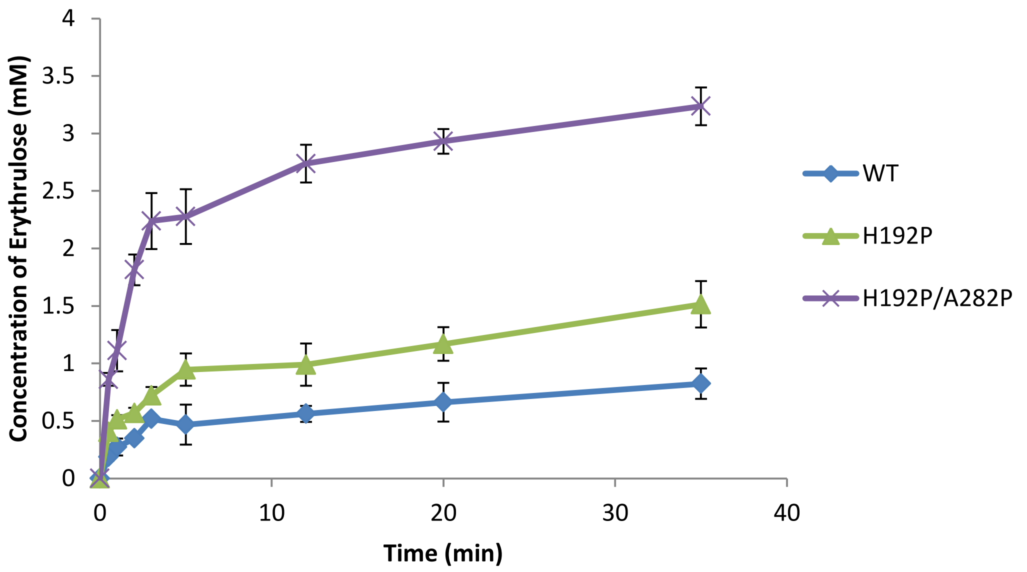

Supplementary Figure S7. Characteristics of TK variants. (A), Unfolding curve of TK mutants triggered by temperature. (B), Natural-logarithmic plot of residual activity vs. length of the heat treatment at 60 °C. WT, y=-0.0303x+4.2627 R^2=0.95; D143K, y=-0.0275x+4.4798 R^2=0.99; I189H, y=-0.0122x=4.71 R^2=0.96; A282P, y=-0.0192x+4.4680 R^2=0.97; H192P, y=-0.0158x+4.6117 R^2=0.93; H192PA282P, y= -0.0110x+4.4969 R^2=0.96. (C), Catalytic activity of TK variants at 60 °C. The catalytic activity was measured in triplicate using 50 mM GA and 50 mM HPA as the substrates in 50 mM Tris-HCl buffer pH 7.0. Specific activities were determined as initial rate/enzyme concentration. (D), Catalytic activity of TK variants at 65 °C. Specific activities of wild type and variants were WT, 3.52 µmol min^{-1} mg^{-1}; H192P, 5.6 µmol min^{-1} mg^{-1}; H192P/A282P, 17.53 µmol min^{-1} mg^{-1}.

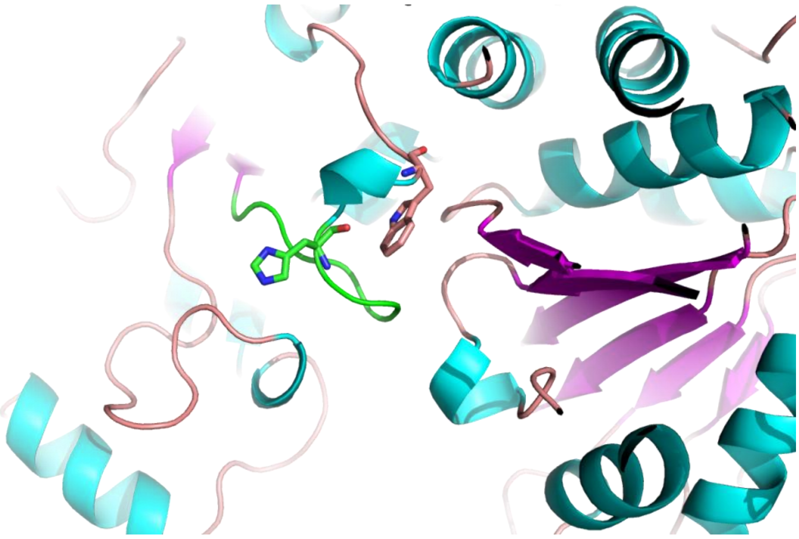

Supplementary Figure S8. 3-D structure of TK showing the positions of loop8 185-192 and Trp196. Green indicates the loop8; His192 and Trp196 were displayed as sticks.

A

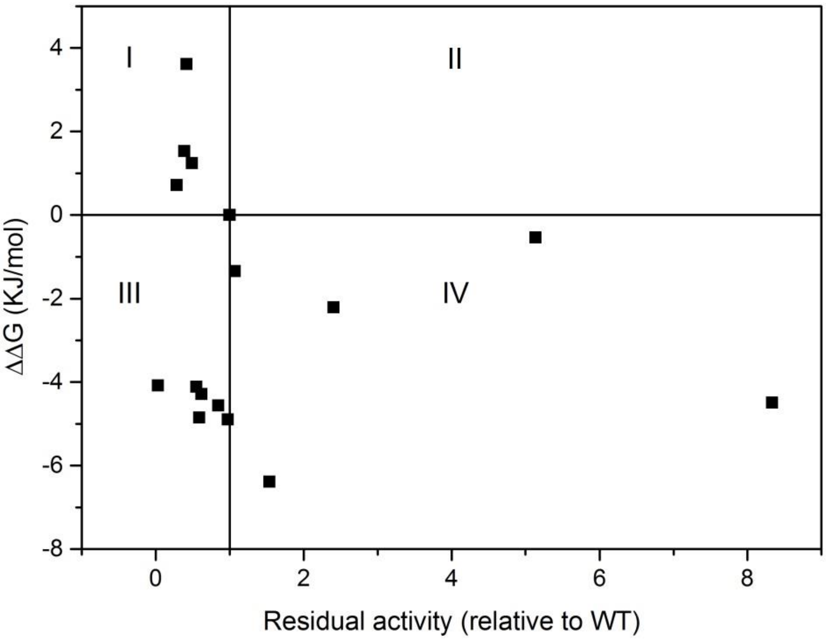

B

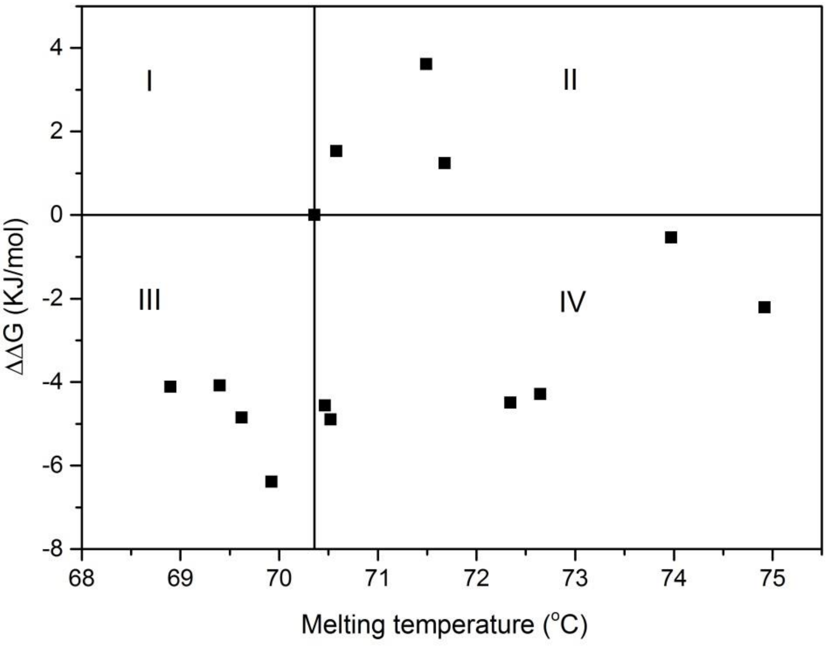

Supplementary Figure S9. Assessment of Rosetta performance. (A), Correlation between ΔΔ*G* values and residual activities of purified TK variants. (B), Correlation between ΔΔ*G* values and T_m of TK variants. 13 variants are I189H, D143K, D146N, D146Y, T245N, K280W, A282E, E332C, H192P, H142C, H142K, H142Q and A282P.

A

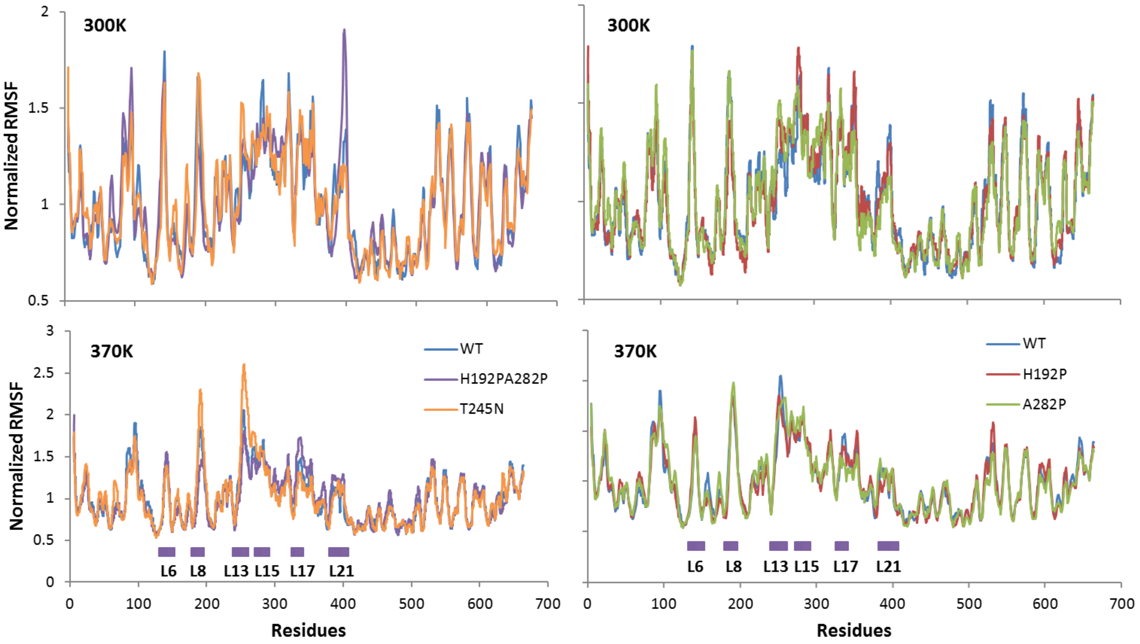
300K
300K
370K
370K
Normalized RMSF
Normalized RMSF
WT
H192PA282P
T245N
WT
H192P
A282P
L6
L8
L13
L15
L17
L21
L6
L8
L13
L15
L17
L21
0
100
200
300
400
500
600
700
Residues
Residues

B

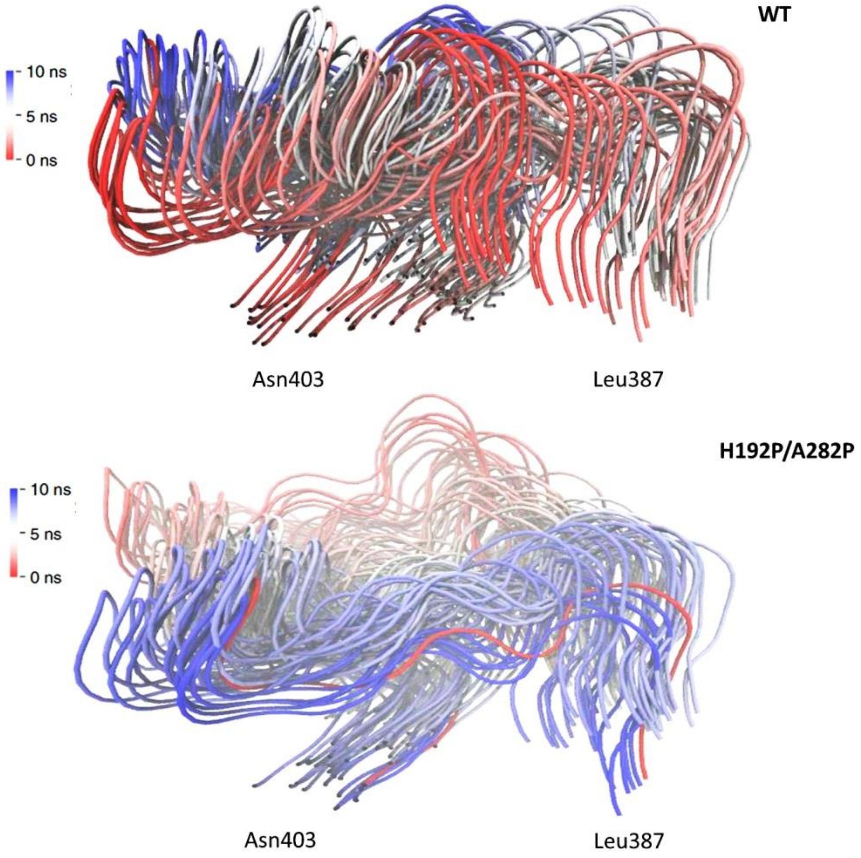
WT
10 ns
5 ns
0 ns
Asn403
Leu387
H192P/A282P
10 ns
5 ns
0 ns
Asn403
Leu387

Supplementary Figure S10. Flexibility of wild-type and mutant TKs. (A), Normalized RMSF values of wild type and variants. The RMSF for the whole protein was calculated with average conformations of last 10-ns trajectory as reference, and normalized RMSF was calculated by dividing the RMSF of each residue by the average of all residues RMSF. (B), Loop21 of H192P/A282P becomes disordered compared to that of WT. For better visibility, only 100 frames within last 10 ns of one MD simulation at 300 K are displayed. Frames at the start simulation are in red, whereas frames at the end are in blue. The pictures were generated by VMD graphics system.

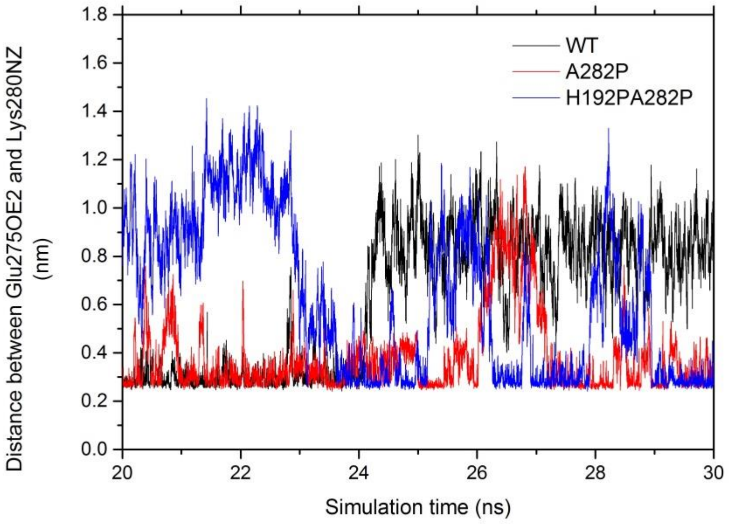

Supplementary Figure S11. The distance between the atom OE2 of Glu275 (Chain A) and the atom NZ of Lys280 (Chain A) changed as the function of simulation time. If the threshold was set 3.2 Å for a salt bridge, then the salt bridge between Glu275 and Lys280 was intact for 3.1 ns, 5.1 ns, 3.2 ns in WT, A282P and H192PA282P respectively throughout the last 10 ns of simulations at 300 K.

Reference

1 Guruprasad, K. & Rajkumar, S. Beta-and gamma-turns in proteins revisited: a new set of amino acid turn-type dependent positional preferences and potentials. *Journal of biosciences* **25**, 143-156 (2000).
